# Para-Methoxybenzylidene Acetal-Protected D-Glucosamine Derivatives as pH-Responsive Gelators and Their Applications for Drug Delivery

**DOI:** 10.3390/gels9060445

**Published:** 2023-05-27

**Authors:** Jonathan Bietsch, Logan Baker, Anna Duffney, Alice Mao, Mary Foutz, Cheandri Ackermann, Guijun Wang

**Affiliations:** Department of Chemistry and Biochemistry, Old Dominion University, Norfolk, VA 23529, USA; jbietsch@odu.edu (J.B.); lbake004@odu.edu (L.B.); aduff003@odu.edu (A.D.); alicemaoa@gmail.com (A.M.); maryolson@vt.edu (M.F.); cacke003@odu.edu (C.A.)

**Keywords:** organogelators, hydrogelators, pH-responsive, carbohydrate, self-assembly, stimuli-responsive

## Abstract

Carbohydrate-based low molecular weight gelators (LMWGs) are compounds with the capability to self-assemble into complex molecular networks within a solvent, leading to solvent immobilization. This process of gel formation depends on noncovalent interactions, including Van der Waals, hydrogen bonding, and π–π stacking. Due to their potential applications in environmental remediation, drug delivery, and tissue engineering, these molecules have emerged as an important area of research. In particular, various 4,6-*O*-benzylidene acetal-protected D-glucosamine derivatives have shown promising gelation abilities. In this study, a series of C-2-carbamate derivatives containing a para-methoxy benzylidene acetal functional group were synthesized and characterized. These compounds exhibited good gelation properties in several organic solvents and aqueous mixtures. Upon removal of the acetal functional group under acidic conditions, a number of deprotected free sugar derivatives were also synthesized. Analysis of these free sugar derivatives revealed two compounds were hydrogelators while their precursors did not form hydrogels. For those protected carbamates that are hydrogelators, removal of the 4,6-protection will result in a more water-soluble compound that produces a transition from gel to solution. Given the ability of these compounds to form gels from solution or solution from gels in situ in response to acidic environments, these compounds may have practical applications as stimuli-responsive gelators in an aqueous medium. In turn, one hydrogelator was studied for the encapsulation and release of naproxen and chloroquine. The hydrogel exhibited sustained drug release over a period of several days, with the release of chloroquine being faster at lower pH due to the acid lability of the gelator molecule. The synthesis, characterization, gelation properties, and studies on drug diffusion are discussed.

## 1. Introduction

Sugar-based low molecular weight gelators (LMWGs) are a class of compounds that have demonstrated a variety of applications over the past decade [1,2,3,4,5]. These compounds form supramolecular gels through non-covalent interactions, producing three-dimensional networks that entrap solvents. Previous research has shown that supramolecular gelators have exhibited a variety of applications in environmental remediation [6,7] ion sensing and chemosensors [8,9] and catalytic reactions [10,11,12]. Stimuli-responsive hydrogelators, especially pH-responsive gelators, are interesting new materials with numerous applications [13,14]. However, a greater likelihood of biocompatibility in carbohydrate-based gelators has led to their resulting gels being studied for potential biomedical applications, including tissue engineering and drug delivery [15,16,17,18,19]. Sugar-based gelators with stimuli-responsive functional groups can therefore form soft materials with combined applications in ion sensing, chemo sensors, and biomedicine [20,21]. Among the many classes of sugar-based gelators, various D-glucosamine derivatives have been demonstrated as materials with numerous potential applications from drug delivery to the discovery of new therapeutic agents and methods [22]. Our group has studied various classes of these sugar derivatives, including the functionalization of glucosamine to form amides, ureas, and carbamates.

**Figure 1:**
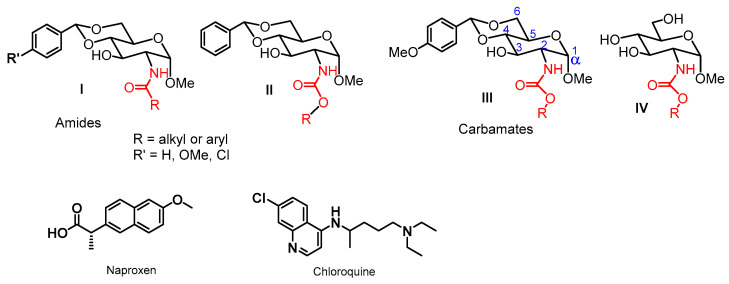
Structure-based design of potential sugar-based LMWGs and the two drug molecules.

In a previous study, we found that various amide (**I**) and several carbamate (**II**) derivatives of 4,6-*p*-methoxybenzylidiene acetal-protected glucosamine form effective gelators (Figure 1) [23,24,25]. These compounds exhibited gelation properties in a variety of solvents, though fewer were hydrogelators in water. Six different carbamate derivatives with the general structure **II** were reported as LMWGs, including two hydrogelators (R = isobutyl and benzoyl) [23]. Introducing the acid labile *p*-methoxy functional group to the phenyl ring did not greatly affect the gelation, although several amide compounds **I** (R′ = OMe) [24] exhibited higher efficiencies in comparison to the parent compound **I** (R′ = H) [26]. Molecular self-assemblies and hydrogels containing stimuli-responsive functionality are important materials for drug delivery and other biomedical applications [27,28]. Systematic studies of different carbamate derivatives using the more acid-sensitive *p*-methoxy benzylidene acetal functional group are important for the discovery of novel effective hydrogelators and organogelators.

In this study, we explore the use of 4,6-*p*-methoxybenzylidene acetal-protected D-glucosamine carbamate derivatives with the general structure **III** as possible LMWGs. A series of different carbamates are synthesized and then analyzed for their gelation properties. Through analysis of structure in relation to their gelation abilities, more effective gelators can be designed. Based on our previous studies, we predict these new series of compounds will form pH-sensitive supramolecular gels [24]. These compounds can be readily converted to their corresponding “free” carbamate derivatives **IV**, and we anticipate that these unprotected glucosamine carbamates may form hydrogels for certain R groups. For compounds that form hydrogels, their protected precursors **III** will be used to study their potential for “pH-instructed self-assembly”. As previously mentioned, these hydrogelators can have various biomedical applications for drug delivery or enzyme immobilization. In this study, two different drugs, naproxen and chloroquine, are used as model drugs due to their unique photophysical properties. Both naproxen and chloroquine drug molecules have distinctive UV–Vis absorptions that can be utilized to monitor the release of the drug from the gel to aqueous phase. Their concentrations can be determined readily by UV–Vis spectrometry with minimal overlapping with the gelator compounds. Naproxen is a nonsteroidal anti-inflammatory drug with a naphthalene acid structure, which can be used for the formation of peptide-like gelators or as model drugs for the study of hydrogel’s capacity of drug encapsulation and sustained release [29,30,31,32,33]. Chloroquine diphosphate is an antimalarial drug used for the treatment of plasmodium falciparum. It is also an autophagy inhibitor that shows an enhanced anticancer effect when used in combination with anticancer drugs [34,35]. Chloroquine has also been used in drug delivery systems through non-covalent or covalent linkages [36,37]. However, the potential for drug delivery via encapsulation in a supramolecular hydrogel has yet to be evaluated.

## 2. Results and Discussion

**Scheme 1:**
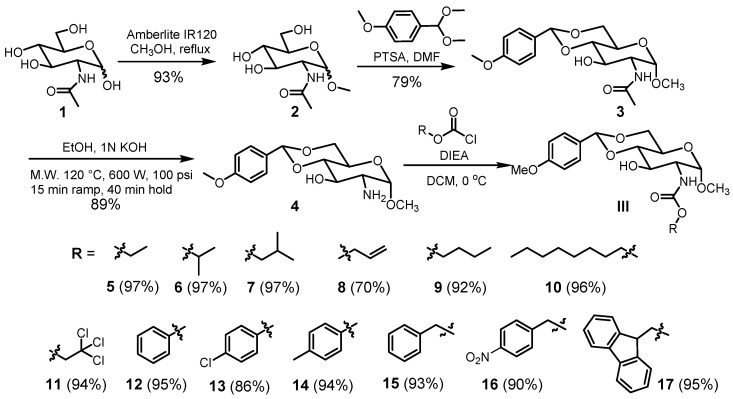
Synthesis of C-2 carbamate derivatives of D-glucosamine.

We synthesized and evaluated the gelation properties of a series of carbamate derivatives that contain an acid labile 4,6-p-methoxylbenzylidene acetal protective group. The synthesis of the carbamate derivative **III** is shown in Scheme 1. Starting with N-acetyl glucosamine **1**, a glycosylation reaction with methanol was carried out to yield compound **2** with the alpha isomer as the major product. The compound was then converted to the p-methoxybenzylidene acetal-protected compound **3**, and the pure alpha isomer was obtained after recrystallization and/or chromatography. Deacetylation of compound **3** under microwave conditions afforded the head group **4**. The amino group in compound **4** was then reacted with various chloroformates to synthesize the final protected carbamate derivatives **5**–**17**. Thirteen different carbamate derivatives with various aliphatic and aromatic groups were synthesized and then analyzed for their gelation properties in a variety of solvents. The results are shown in Table 1.

**Scheme 2:**
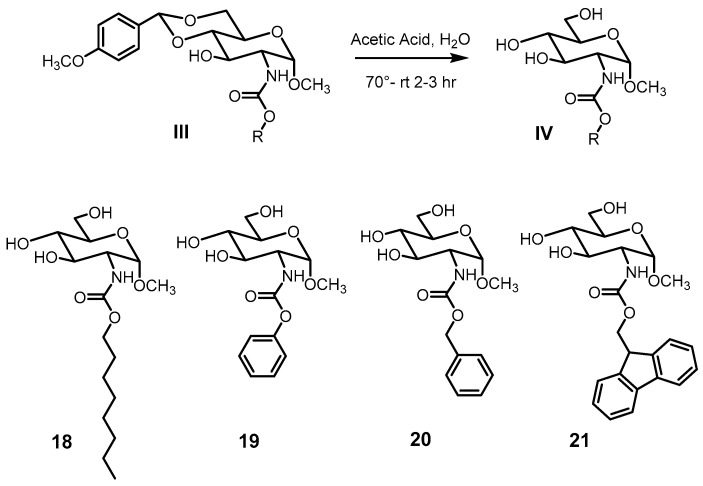
Structures of the deprotected glucosamine carbamate derivatives.

Four compounds, including a long-chain alkyl and three aryl derivatives, were selected to prepare the unprotected carbamate derivatives **IV** (**19**–**22**), as shown in Scheme 2. Their gelation properties are also tested and included in Table 1.

These compounds exhibited excellent gelation properties in many solvents, including organic solvents, alcohols, and aqueous mixtures. In general, short-chain linear alkyl derivatives exhibited good gelation properties in several solvents, including water. The ethyl derivative **5** and isopropyl derivative **6** formed gels in 11 different selected test solvents. The four-carbon branched compound **7**, the isobutyl derivative formed gels in nine different solvents. The allyl derivative **8** formed gels in six solvent systems, relatively fewer solvents compared to the other three compounds. All four short-chain carbamates formed gels in ethanol, ethylene glycol, ethanol/water mixtures and DMSO/water 1:1 mixture. Compounds **5** and **7** also formed hydrogels at 5.0 and 1.5 mg/mL, respectively. The five carbon linear alkyl derivative **9** only formed gels in five different solvent systems but it was one of the most effective hydrogelators in this study, forming a hydrogel at 0.9 mg/mL. It also formed gels in EtOH/water and DMSO/water mixed solvents. Further increasing the chain length to octyl derivative **10** resulted in gelation in three solvents, fewer in comparison to the other aliphatic derivatives. The octyl derivative compound **10** formed gels at 10 mg/mL in hexane, glycerol, and triethylene glycol. The trichloroethyl derivative **11** performed inferiorly compared to the isobutyl derivative **7**. The trichloro derivative formed gels at higher concentrations typically. This compound has a bulky CCl_3_ functional group and caused more steric repulsion compared to the other short-chain alky derivative without the chloro substitution. The aromatic derivatives showed a diverse range of gelation behaviors. The phenyl carbamate **12** formed gels in 10 different solvents; however, the parasubstituted phenyl derivatives **13** and **14** were not gelators for most of the tested solvents. The trend reflected the sensitivity of the functionality in the compounds and additional nonpolar functional groups are not beneficial for gelation. Similarly, the benzyl derivative **15** is a versatile gelator, which formed gels in nine different solvents. The paranitro substituted benzyl derivative **16**; on the other hand, it didn’t form gels in these solvents. Interestingly, when the bulky FMoc functionality was introduced, the carbamate **17** turned out to be an excellent gelator, forming gels in **10** different solvents, with several forming at low concentrations.

**Figure 2:**
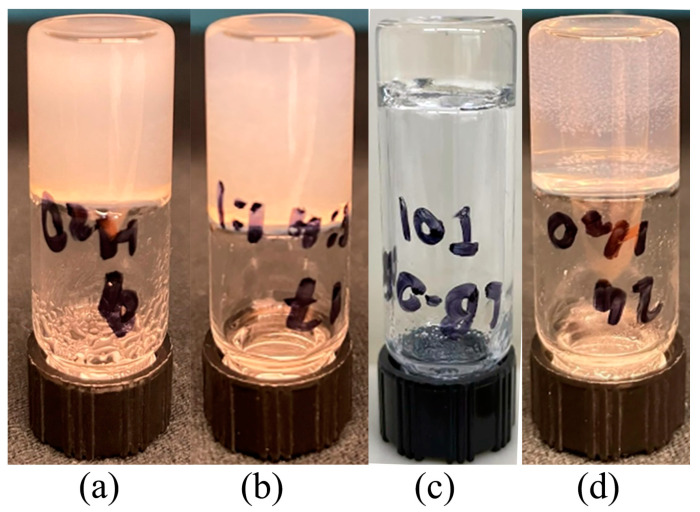
Gel photos for several gelators. (**a**) A hydrogel formed by compound **9** at 0.9 mg/mL; (**b**) a gel formed by compound **17** at 0. 8 mg/mL in EtOH:H_2_O (1:1); (**c**) a clear gel formed by compound **20** in toluene at 2.9 mg/mL; (**d**) a clear to translucent gel formed by compound **21** in water at 0.9 mg/mL.

In triethylene glycol, compound **10** formed a gel at 10.0 mg/mL and compound **13** formed a gel at 20.0 mg/mL; all other compounds were soluble. In hexanes, compound **10** formed a gel at 10.0 mg/mL, and compounds **6**, **16**, and **18** formed precipitates; all other compounds were insoluble. A majority of the gels appeared opaque and sometimes translucent to opaque. The toluene gels for the deprotected sugar derivatives typically are clear or transparent. The compound **21** also formed a clear gel in chloroform. Some gel photos are included in Figure 2.

From Table 1, three aliphatic carbamate derivatives (**5**, **7**, **9**) formed hydrogels, while most aromatic carbamate derivatives were insoluble in water. We hypothesized that the removal of the 4,6-benzylidene acetal functional group to form the free sugar carbamate derivative **IV** could increase solubility in aqueous solutions and lead to more effective hydrogelators. Therefore, four compounds (octyl carbamate **10**, phenyl carbamate **12**, benzyl carbamate **15**, and Fmoc carbamate **17**) were selected to be converted into free sugar carbamate derivatives. When analyzing gelation properties, we compared the 4,6-protected carbamate to their respective deprotected “free” sugar carbamate to gain insight into the relationship between structure and gelation capability. For compound **10**, the benzylidene acetal group and the long alkyl chain resulted in a compound with both π–π interactions and hydrophobic functions to perform as an effective gelator in alcohols and aqueous mixtures. Removal of the acetal protection to form compound **18** encouraged gelation properties in water, aqueous mixtures, and toluene. For phenyl carbamate **12** and benzyl carbamate **15**, the 4,6-benzylidene acetal played important roles in gelation. The protected derivatives formed gels in 10 different solvents, while the deprotected derivatives did not form gels in any of the solvents. The Fmoc derivative **17** is an effective gelator with a significant amount of aromatic functionality. Fmoc derivatives have exhibited excellent gelation properties and biological activities [38,39,40]. The protected derivative **17** formed gels in 10 different solvents and at low concentrations. Interestingly, the deprotected Fmoc derivative **21** was also observed as an effective gelator. It formed gels in twelve different solvents and formed a hydrogel at 0.9 mg/mL. These comparisons indicate that the balance between intermolecular interactions is very important to molecular assembly in different solvent systems. In general, the 4,6-p-methoxyl benzylidene acetal derivatives are effective gelators and can be used as a template for designing new LMWGs. The deprotected free sugar carbamate derivatives, in particular, require longer alkyl chains or bulky aromatic functional groups attached to the C-2 position to achieve gelation. However, these highly polar sugar derivatives are more effective at forming hydrogels and have the potential for various biomedical applications.

**Figure 3:**
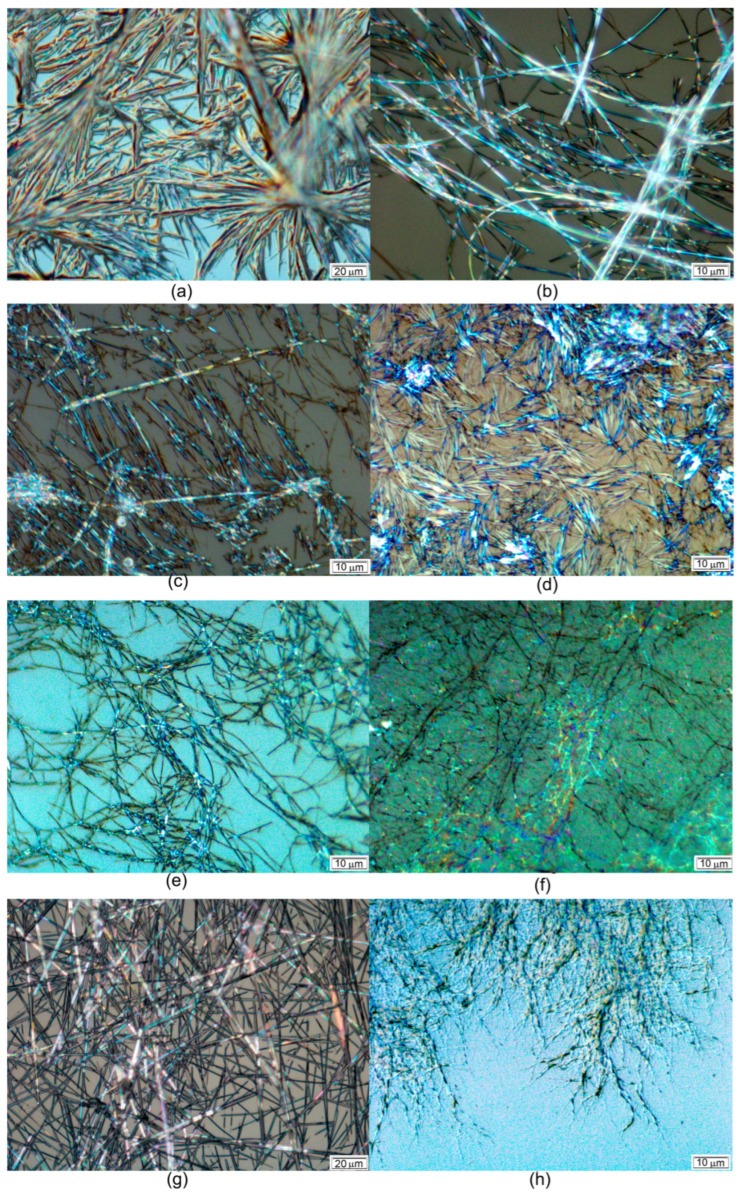
Optical micrographs of the gels formed by several compounds: (**a**) Compound **5** in water at 5.0 mg/mL; (**b**) Compound **7** in water at 1.5 mg/mL; (**c**) Compound **9** in PBS buffer 2.0 mg/mL; (**d**) Compound **15** in DMSO:H_2_O (*v*/*v*, 1:2) at 6.7 mg/mL; (**e**) Compound **17** in EtOH:H_2_O (*v*/*v* 1:1) at 0.8 mg/mL; (**f**) Compound **18** in toluene at 3.3 mg/mL; (**g**) Compound **18** in water at 10.0 mg/mL; (**h**) Compound **21** in water at 1.0 mg/mL.

The optical micrographs of several representative gels are shown in Figure 3. Due to their potential biomedical applications, we prioritized the characterization of the hydrogelators. The gel morphology primarily exhibited fibrous-like features. The hydrogel formed by compound **5** in water at 5.0 mg/mL is composed of bundled straight fibers, as shown in Figure 3a. A small amount of solvent is still present and can be observed in the image. The hydrogel formed by compound **7** at 1.5 mg/mL is composed of a long, uniform, fibrous network. The fibers in this network appear curved and have a smaller diameter than the fibers shown in Figure 3a. The butyl derivative **9** formed a hydrogel at 0.9 mg/mL and exhibited a uniform fibrous network with more helictical features. The smaller fibers were difficult to visualize under an optical microscope due to their smaller diameter. We also prepared a hydrogel of this compound in PBS buffer. The morphology of the gel also appeared as long curved fibers (Figure 3c). The benzyl derivative compound **15** in DMSO:H_2_O (*v*/*v* 1:2) at 6.7 mg/mL appeared as relatively shorter bundles of fibers, uniform in width and length (Figure 3d). The Fmoc derivative compound **17** was a versatile gelator; its morphology in EtOH:H_2_O (*v*/*v* 1:1) at 0.8 mg/mL exhibited long tubular fibers with uniform small diameters and curvatures (Figure 3e). The deprotected free sugar carbamate **19** formed gels in toluene, and its fibers appeared to be uniform, curved long fibers with a small diameter and multiple branching points (Figure 3f). The hydrogel formed by the deprotected octyl carbamate **18** at 10.0 mg/mL showed long, straight fibers with fewer curvatures, with large tubules occasionally bundled to form larger planar sheets (Figure 3g). The deprotected Fmoc derivative compound **21** formed hydrogels at much lower concentrations (0.1 wt % in water). The morphology showed thin and long fibrous networks with many branching points (Figure 3h).

**Figure 4:**
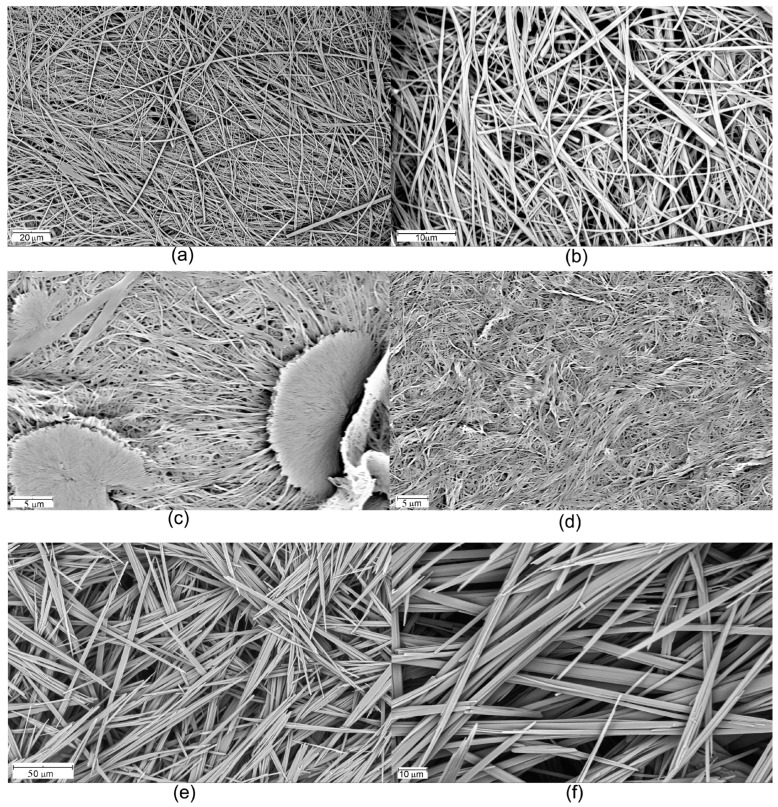
SEMs of the xero gels of hydrogels from (**a**,**b**): compound **7** at 1.5 mg/mL; (**c**,**d**): compound **9** at 0.9 mg/mL; (**e**,**f**): compound **18** at 10.0 mg/mL.

The hydrogels formed by compounds **7** and **9** were also characterized using scanning electron microscopy. A few representative scanning electron micrographs (SEMs) are shown in Figure 4. The hydrogel of **7** formed long fibers with some curvature. In certain sections, the fibers formed bundles to create planar ribbons or sheets (Figure 4a,b). The hydrogel of compound **9**, on the other hand, formed a more uniform fibrous network with more curved and helical features. In more concentrated regions they appear as more densely packed disks composed of smaller fibers (Figure 4b,c). The deprotected octyl carbamate compound **18** formed a hydrogel at 10 mg/mL. The morphology of the dried gel appears as straight fibers with relatively shorter lengths (Figure 4e,f). In this study, our most efficient gelator, compound **9**, formed a hydrogel at 0.9 mg/mL. Its gel morphology exhibited a thin and circular fibrous network with a uniform diameter and distribution, as shown in Figure 4c,d. The isobutyl derivative compound **7** required a slightly higher gelation concentration of 1.5 mg/mL. The gel appeared as relatively larger fibers, compared to those of compound **9**, and was composed of long fibers with specific curvatures (Figure 4a,b). Compound **18** required a gel concentration of 10 mg/mL in water. The gel mainly formed short and straight fibers, with the capability of forming bundles (Figure 4e,f). It is interesting to note that there is a correlation between the morphology and the MGCs of the gels. Hydrogelators with lower MGCs tend to form narrow and uniform entangled fibrous networks, while those with higher MGCs tend to form larger planar sheets, ribbons, or cylindrical tubules.

### 2.1. Drug Delivery Studies

**Figure 5:**
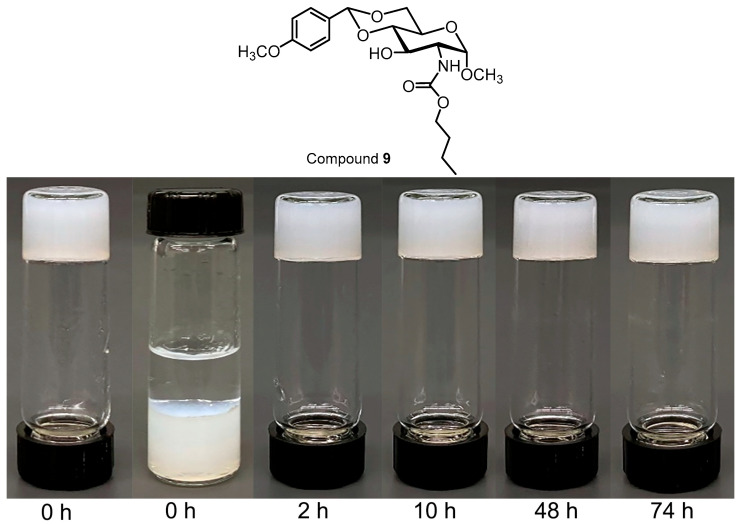
Gel photographs of compound **9** with naproxen in the initial condition (0 h), before and after adding 2 mL water, followed by the gel photos at selected time points after the aqueous phase was removed.

The n-butyl carbamate compound **9** demonstrated efficient hydrogelation properties by forming a hydrogel at a low concentration of 0.9 mg/mL. This compound was selected for further drug delivery studies using the anti-inflammatory drug naproxen and the antimalarial drug chloroquine. The hydrogels formed by the drug molecules in combination with compound **9** (2.0 mg/mL gel) were prepared in a vial. Then, 2.0 mL water or buffer solution was added to the top of the gel. The amount of naproxen that diffused from the gel to the liquid phase was monitored by removing the aqueous phase at set times and measuring the UV–Vis absorption of the aqueous phase. Figure 5 presents photographs of hydrogelator **9** with naproxen sodium at different time points. The gel appeared stable for several days, as shown in the photo.

**Figure 6:**
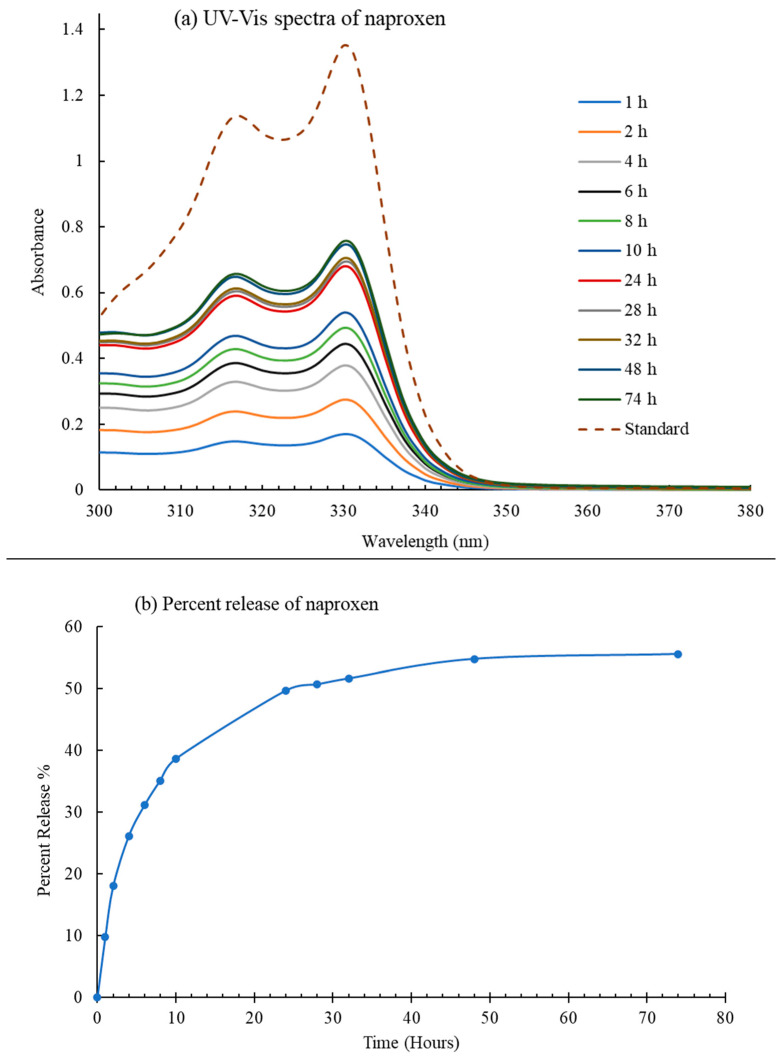
The UV–Vis spectra (**a**) and percent release profile (**b**) of naproxen sodium over time from a co-gel formed by compound **9** (2.0 mg/mL gel) with 0.25 mg/mL naproxen sodium in H_2_O.

The timed-release UV–Vis spectra and estimated percent release profile for naproxen sodium are shown in Figure 6. The gel was prepared using 4.0 mg of compound **9** and 0.5 mg of naproxen sodium in 2.0 mL water. The standard shown in the spectra for naproxen sodium is 0.25 mg/mL (0.991 mM). The percent release was calculated using absorption values at λ = 331 nm for each time point, and the ratio to the standard was taken and plotted in Figure 6b. As shown in Figure 6, the naproxen concentration approached 50% (the maximum) at 24 h, which indicated that the naproxen in the aqueous phase and gel phase had reached equilibrium. Further measurement over a longer period revealed slightly over 50% release due to the possibility of hydrogel shrinkage over time.

In addition to naproxen, we selected the antimalarial drug chloroquine diphosphate to further study the hydrogel’s capabilities. Chloroquine can be protonated at lower pHs and exhibits greater water solubility at those pH values than at neutral pH. Additionally, there has not been a small molecular gelator reported to form a two-component gel with chloroquine. Due to chloroquine’s importance in different therapeutic areas, we investigated the ability of hydrogelator **9** to form a co-gel with the drug molecule. Our findings demonstrated that the hydrogelator was capable of forming a gel in water in combination with chloroquine, enabling us to proceed with a drug delivery study similar to the naproxen study.

**Figure 7:**
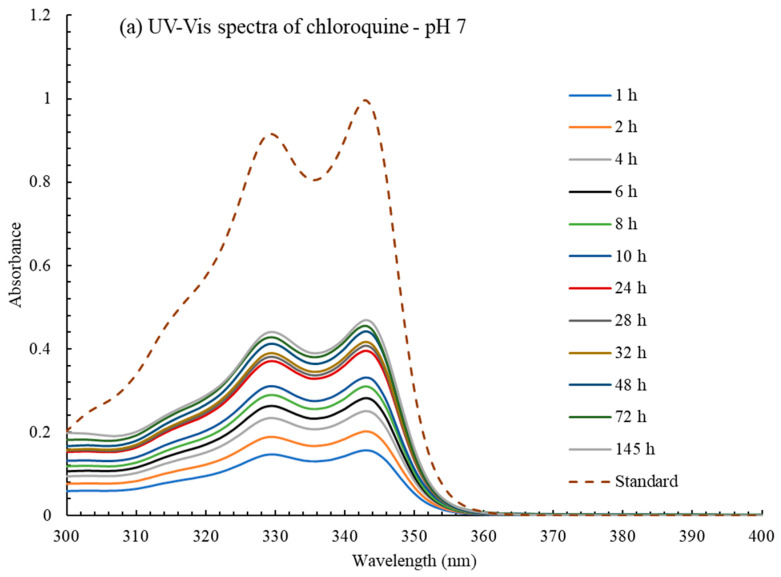

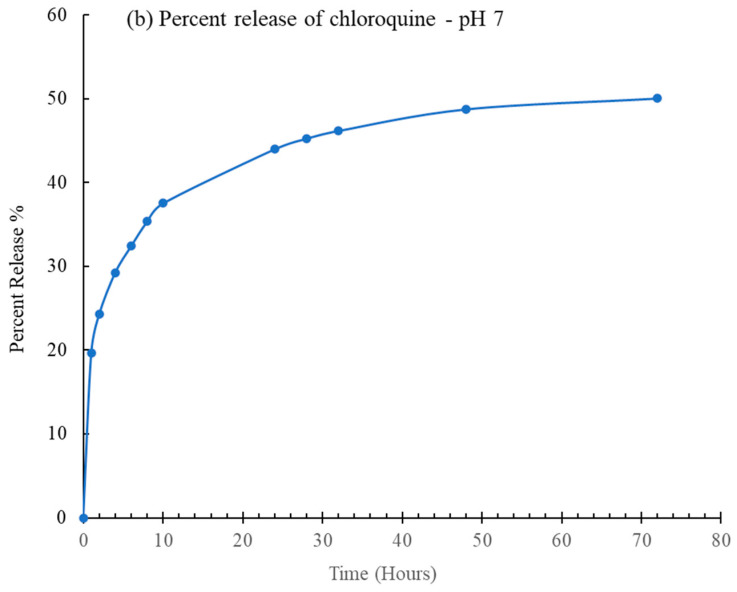
The UV–Vis spectra (**a**) and percent release profiles (**b**) of chloroquine diphosphate to a pH 7.0 aqueous phase over time from co-gels formed by compound **9** (2.0 mg/mL gels). Percent release was calculated using absorption values at 343 nm for each time point versus the standard.

**Figure 8:**
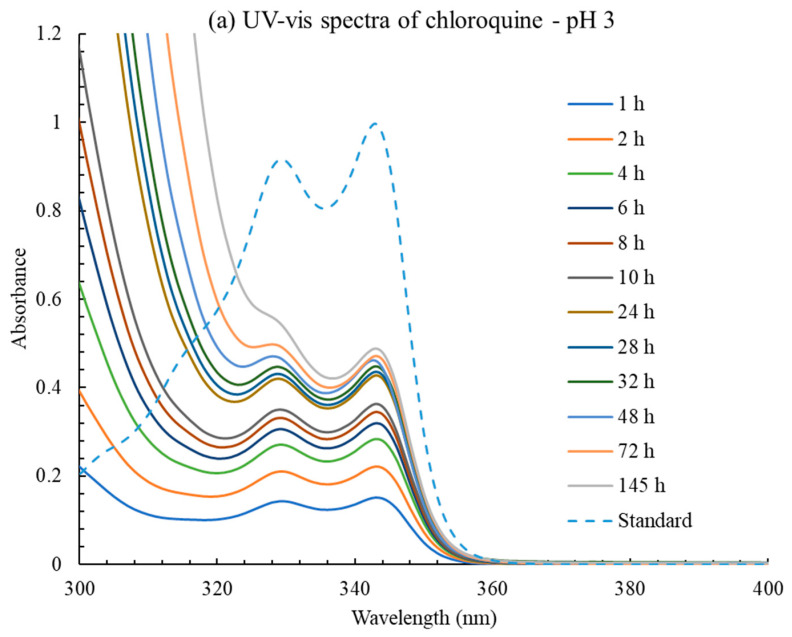

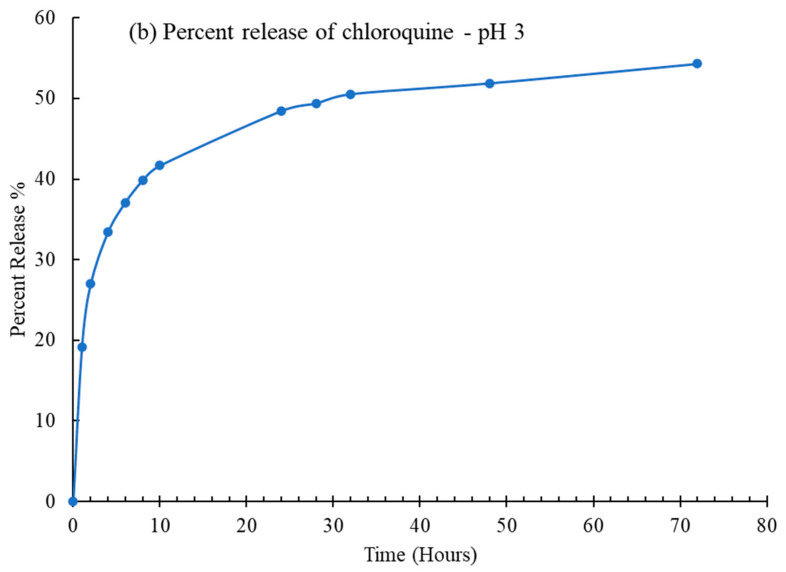
The UV–Vis spectra (**a**) and percent release profiles (**b**) to a pH 3.0 aqueous phase of chloroquine diphosphate over time from the co-gels formed by compound **9** (2.0 mg/mL gels) in H_2_O. Others are the same as Figure 7.

A hydrogel was prepared using 4.0 mg of compound **9** and 0.05 mg of chloroquine diphosphate in 2.0 mL DI water. The initial concentration of the gelator in the gel was 2.0 mg/mL, and the chloroquine concentration was 0.025 mg/mL. The concentration of chloroquine was selected based on a UV calibration curve, where the maximum absorption was maintained at below 1. To this gel, 2.0 mL of an aqueous buffer was added on top; these aqueous phases were prepared using the McIlvaine buffer system at different pHs. The aqueous phase was removed from the top of the gel, and the UV–Vis spectra were obtained at different time points, as shown in Figure 7 and Figure 8. The chloroquine diphosphate standard concentration was 0.025 mg/mL (0.0485 mM). Therefore, if the gel was sufficiently stable at equilibrium, 50% release of the drug from the gel phase to the aqueous phase could be achieved; this would be considered equal to 100% diffusion of the drug molecules. The ratio of the UV absorption at 343 nm for the aqueous phase to the standard absorption is shown in Figure 7b and Figure 8b. pH 7 buffer and pH 3 buffer were used in Figure 7 and Figure 8, respectively. Both graphs show that chloroquine release at pH7 reached a maximum at approximately 72 h, with a slightly higher maximum observed at 145 h. Compared to the pH 7 study, the percentage of drug released at pH 3 was only slightly higher. A more drastic change can be achieved using stronger acidic conditions, which enable the gelator degradation much more rapidly. The photographs of the gel at various time points can be found in the Supplementary Materials.

### 2.2. Acid-Triggering Gelation Studies

The drug encapsulation and diffusion studies shown above indicated that the hydrogels are stable for several days at neutral pHs and mild acid conditions at room temperature (rt). Since compound **18** formed a hydrogel, but the precursor **10** is not a hydrogelator, to determine if the gels could undergo self-assembly in situ in acidic conditions, we conducted tests to assess gel formation under such conditions. Compound **10** was tested at pH 1 and pH 4; after 24 h at rt there was no gelation; however, with gentle heating, both samples formed gels. To test the acid-triggered gelation, compound **10** was added to a vial along with pH 1 solution. After incubating in a shaker at 37 °C for 72 h, gelation was observed (Supplementary Figure S26). Small samples were placed on a microscope slide and visualized via optical microscopy, exhibiting a morphology with long straight fibers (Figure 3g). Additional photos of the gels are included in the Supplementary Figure S27. This study confirms that the p-methoxybenzylidene-protected sugar carbamate derivatives can be converted to hydrogels under acidic conditions.

## 3. Conclusions

In summary, we have designed and synthesized a library of sugar-based LMWGs, consisting of 13 different *p*-methoxy substituted benzylidene acetal functionalized glucosamine 2-carbamates. All aliphatic derivatives are effective gelators for at least one of the selected solvents. Among the aromatic derivatives, the phenyl, benzyl, and Fmoc derivatives are the most efficient gelators, forming gels in at least nine of the tested solvents. The octyl, phenyl, benzyl, and Fmoc derivatives were treated with acid to form their respective deprotected free sugar carbamate derivatives. All deprotected derivatives show gelation in toluene. The Fmoc derivative **21** is the most versatile gelator among all carbamates, forming gels in 12 different solvents and a hydrogel at 0.09 wt %. The deprotected octyl derivative **18** formed a hydrogel at 1.0 wt %, while the protected octyl derivative was not a hydrogelator. Treating compound **10** with acidic aqueous solution resulted in a hydrogel formation, demonstrating an acid-triggered gelation response. Hydrogelator compound **9**, which formed a hydrogel at 0.09 wt %, was used for drug encapsulation and diffusion studies. It formed gels in the presence of naproxen sodium and chloroquine and showed sustained release over a period of several days. For chloroquine, lower pH resulted in a slightly faster release of the drug to the aqueous phase. These new gelator compounds, specifically the hydrogelator compounds, have the potential for future applications in biomedical fields.

## 4. Materials and Methods

General method and materials: All reagents and solvents were purchased from chemical suppliers and used as they were received. Purifications were carried out through recrystallization, trituration, or column chromatography using 230–400 mesh silica gel using gradient solvent systems. A Bruker 400 MHz spectrometer (Bruker, Billerica, MA, USA) was utilized to obtain ^1^H NMR, proton-decoupled ^13^C NMR, HSQC, and COSY in either CDCl_3_ or DMSO-*d*_6_. The chemical shifts reported are based on using CDCl_3_/DMSO-*d*_6_ as internal standards at 7.26/2.50 ppm (^1^H NMR) and at 77.00/39.50 ppm (^13^C NMR), respectively. Melting point analysis was carried out using a Fisher Jones melting point apparatus. The molecular mass was measured using LCMS on an Agilent 6120B Single Quad Mass Spectrometer (Agilent, Santa Clara, CA, USA) and LC1260 system or Shimadzu LCMS-2020 (Shimadzu, Canby, OR, USA) with ESI in positive ionization mode.

Optical Microscopy: Olympus BX60M optical microscope, equipped with an Olympus DP73-1-51 high-performance Peltier cooled 17 MP digital camera with pixel shifting, was used to analyze the morphology of gel samples. Gel samples were placed on clean glass slides and allowed to air dry before analysis. Images were captured using CellSens 1.11 computer software.

Scanning Electron Microscopy: SEM images were obtained using a Thermo Scientific Phenom XL G2 Desktop SEM instrument. The gel sample was placed on the sample holder button and air dried for 5–7 days and then stored in a nitrogen flushed desiccator for about 7 days. The dried samples were coated with silver for about 30–60 s before analysis.

Gel Testing: All experiments were started at 20 mg/mL by measuring out about 2 mg of compound in a 1 dram vial and 0.1 mL of the solvent. The sample was then heated gently until the compound dissolved, forming a homogeneous solution. The solution was then allowed to cool to room temperature and sit undisturbed for 30 min. The vial inversion method was used to determine gel formation, where an inverted vial containing a gel has no flow under gravity. Serial dilutions were carried out on all gels that were found to be stable at 20 mg/mL until the minimum gelation concentration was discovered.

### 4.1. Naproxen Trapping and Release Studies

A stock solution was prepared by dissolving 2.5 mg of naproxen sodium in 10.0 mL of DI water. Gels were prepared by heating 4.0 mg of compound **9** with 2.0 mL of the naproxen sodium stock solution, and were then left to stand at room temperature for ~24 h. Then, 2.0 mL of DI water at pH 7 was placed on top of the gel. At specific time intervals, the aqueous phase was carefully removed and transferred to a quartz cuvette to record the UV absorbance and then returned to the gel vial. The gelator concentrations were 2.0 mg/mL, and the initial naproxen sodium concentration was 0.25 mg/mL.

### 4.2. Chloroquine Trapping and Release Studies

A stock solution was prepared by dissolving 2.0 mg of chloroquine diphosphate salt in 10.0 mL of DI water, then serial diluted to obtain a 0.0485 mM (0.025 mg/mL) solution. Gels were prepared by heating 4.0 mg of compound **9** with 2.0 mL of the chloroquine diphosphate solution (0.025 mg/mL) and were then left to sit at room temperature for ~24 h. Two diffusion studies were conducted at different pHs to analyze the effects of aqueous phase pH on the drug release rate. Using the McIlvaine pH buffer system, solutions of pH 7.0 and pH 3.0 were prepared by mixing set volumes of aqueous 0.2 M sodium phosphate dibasic and 0.1 M citric acid solutions. Then, 2.0 mL of pH 7.0 or pH 3.0 McIlvaine buffer was placed on top of the gel. At specific time intervals, the aqueous phase was carefully removed and transferred to a quartz cuvette to record the UV absorbance and then returned to the gel vial. The gelator concentrations were 2.0 mg/mL, and the initial chloroquine diphosphate concentration was 0.025 mg/mL.

### 4.3. General Procedure for the Preparation of Carbamate Compounds ***3***–***4***

Synthesis of Compound **3**. The preparation followed the literature procedure [18]. *P*-methoxybenzaldehyde (1.239 mL, 10.2 mmol, 1.2 equiv), trimethyl orthoformate (2.08 mL, 19.12 mmol, 2.25 equiv), pTSA (162 mg, 0.850 mmol, 0.1 equiv), and anhydrous methanol (10 mL) were added to a dried and nitrogen flushed 50 mL round-bottomed flask. The flask was equipped with a condenser, and the reaction mixture was heated to reflux. After 90 min, the solvent was removed under reduced pressure. Compound **2** was dried through co-distillation with toluene (15 mL), dissolved in anhydrous DMF (5 mL), and added to the reaction mixture. The reaction mixture was then heated to 80 °C for 5 h. The reaction mixture was cooled to rt and quenched with saturated NaHCO_3_ solution (5 mL). The reaction mixture was poured into 50 mL of DI water, causing the product to precipitate. The precipitate was filtered, and the filtrate was extracted with DCM (50 mL × 3). The organic layers were washed with DI water (50 mL × 2), dried over Na_2_SO_4_, filtered, and the solvent was removed under reduced pressure. The crude product was recrystallized in ethanol. The mother liquor was purified via column chromatography on silica gel using pure DCM to 5% MeOH/DCM (NH_3_). The compound **3** was obtained as a white solid, yield 2.24 g (75%). R_f_ = 0.2 in 3% MeOH/DCM. ^1^H NMR (400 MHz, CDCl_3_) *δ* 7.41 (d, *J* = 8.7, 2H), 6.88 (d, *J* = 8.7, 2H), 5.91 (d, *J* = 8.6 Hz, 1H), 5.51 (s, 1H), 4.71 (d, *J* = 3.8 Hz, 1H), 4.15–4.13 (m, 2H), 3.88 (t, *J* = 9.5, 1H), 3.79 (s, 3H), 3.70–3.78 (m, 2H), 3.55 (t, *J* = 9.0 Hz, 1H), 3.39 (s, 3H), 3.18 (s, 1H), 2.04 (s, 3H). ^13^C NMR (100 MHz, CDCl_3_) *δ* 171.4, 160.2, 129.6, 127.6, 113.6, 101.9, 98.8, 82.0, 70.6, 68.8, 62.4, 55.3, 55.3, 54.1, 23.3.

Synthesis of Compound **4**. Compound **4** was prepared according to the literature procedure [18]. Compound **3** (2.00g, mmol, 1 equiv) was added to two EasyPrep microwave vessels. 1N NaOH ethanol solution (50 mL) was added to each vessel. The reaction was carried out under microwave conditions at 100 psi, 600 W, and 120 °C for a 15 min ramp and a 45 min hold time. EtOH was removed under reduced pressure leaving a yellow solid. The solid was dissolved in 30 mL DCM and washed with DI water (30 mL × 3). Aqueous layers were extracted with DCM (30 mL × 2). Organic layers were combined, dried over Na_2_SO_4_, filtered, and the solvent was removed under reduced pressure. The crude product was purified via column chromatography on silica gel using 0–7% MeOH/DCM (NH_3_), producing a white solid as the pure product, yield 1.605 g (90.5%). R_f_ = 0.1 in 5% MeOH/DCM. mp = 179.5–180.0 °C. ^1^H NMR (400 MHz, CDCl_3_) *δ* 7.38–7.45 (m, 2H), 6.86–6.92 (m, 2H), 5.50 (s, 1H), 4.68 (d, *J* = 3.5 Hz, 1H), 4.23–4.28 (m, 1H), 3.68–3.84 (m, 6H), 3.45 (t, *J* = 9.1 Hz, 1H), 3.41 (s, 3H), 2.76–2.83 (m, 1H). ^13^C NMR (100 MHz, CDCl_3_) *δ* 160.2, 129.8, 127.6, 113.7, 101.8, 101.3, 82.0, 71.9, 69.1, 62.6, 56.7, 55.4, 55.3.

### 4.4. General Procedure for the Preparation of Carbamate Compounds ***5***–***17***

All carbamate derivatives were synthesized using the corresponding chloroformates. Compound **4** (1 equiv) was dissolved in anhydrous DCM in a dried 50 mL round-bottomed flask under nitrogen atmosphere. Diisopropylethylamine (DIEA) (3 equiv) was then added to the reaction mixture, and the temperature was reduced to 0 °C via an ice bath. The respective chloroformates were dissolved in anhydrous DCM (2 mL) and added dropwise to the reaction mixture. After 10 min of stirring at 0 °C, the ice baths were removed, and the reaction temperature warmed to rt. Reaction time typically ranged from 1–4 h. Reaction monitoring was carried out through ^1^H NMR and TLC. At the completion of the reaction, the reaction mixture was diluted with DCM (15 mL) and washed with DI water (15 mL × 2). The aqueous layers were back extracted with DCM (15 mL). The combined organic layers were dried over Na_2_SO_4_, filtered, and solvent was removed under reduced pressure. The crude products were purified through column chromatography on silica gel using a gradient solvent system. Each column was treated with a small amount of 0.1 N NH_3_ in methanol/DCM to prevent deprotection of the desired product. The detailed preparation for compound **5** is provided below, and only amounts used and characterization data are provided for all other compounds. All compounds were synthesized using 100 mg (0.32 mmol) of compound **4**.

Synthesis of Compound **5**. Compound **4** (0.100 g, 0.32 mmol, 1 equiv) was dissolved in 5 mL of anhydrous DCM and added to a dried and nitrogen-flushed 50 mL round-bottomed flask. DIEA (0.166 mL, 0.95 mmol, 3 equiv) was added to the flask and the temperature was reduced to 0 °C via an ice bath. Ethyl Chloroformate (33 µL, 0.34 mmol, 1.05 equiv) was dissolved in 2 mL of anhydrous DCM and added dropwise to the reaction mixture over 30 min. The reaction was stirred at 0 °C for 10 min and the ice bath was removed to allow the reaction mixture to warm to room temperature. The reaction stirred at rt for 2 h, at which point it was diluted with DCM (15 mL) and extracted with DI water (15 mL × 2). The aqueous layer was back extracted with DCM (15 mL). Organic layers were dried over Na_2_SO_4_, filtered, and the solvent was removed under reduced pressure. The crude product was purified via column chromatography on silica gel using 0–2% MeOH/DCM, the methanol contains 0.1N ammonium, producing a white solid. Yield: 118 mg (96.7%). R_f_ = 0.4 in 3% MeOH/DCM. mp = 142.5–145.0 °C. ^1^H NMR (400 MHz, CDCl_3_) *δ* 7.38–7.44 (m, 2H); 6.85–6.91 (m, 2H); 5.50 (s, 1H); 5.05 (d, *J* = 6.5 Hz, 1H); 4.72 (d, *J* = 3.3 Hz, 1H); 4.20–4.30 (m, 1H); 4.15 (q, *J* = 7.10 Hz, 2H); 3.82–3.96 (m, 2H); 3.69–3.81 (m, 5H); 3.54 (t, *J* = 8.9 Hz, 1H); 3.39 (s, 3H); 2.84 (s, 1H); 1.26 (t, *J* = 7.1 Hz, 3H). ^13^C NMR (100 MHz, CDCl_3_) *δ* 160.2, 157.0, 129.6, 127.6, 113.7, 101.8, 99.2, 81.9, 70.5, 68.8, 62.4, 61.4, 55.5, 55.3 (d), 14.5. LC-MS m/z calculated for C_18_H_26_NO_8_ [M+H]^+^ 384.40; mass found 384.

Synthesis of Compound **6**. Isopropyl chloroformate (38.3 µL, 0.34 mmol, 1.1 equiv) was reacted with compound **4** (100 mg, 1.0 eq, 0.32 mmol) in DCM with DIEA. The reaction was stopped at 1 h. The crude product was purified via column chromatography on silica gel using 0–5% MeOH/DCM (NH_3_) to yield a white solid. Yield: 125 mg (98%). R_f_ = 0.5 in 3% MeOH/DCM. mp = 162.5–165.0 °C. ^1^H NMR (400 MHz, CDCl_3_) *δ* 7.38–7.44 (m, 2H), 6.86–6.91 (m, 2H), 5.51 (s, 1H), 4.86–5.08 (m, 2H), 4.73 (d, *J* = 3.2 Hz, 1H), 4.20–4.31 (m, 1H), 3.82–4.00 (m, 2H), 3.80 (s, 3H), 3.70–3.79 (m, 2H), 3.54 (m, 1H), 3.40 (s, 3H), 1.25 (d, *J* = 6.3, 6H). ^13^C NMR (100 MHz, CDCl_3_) *δ* 160.2, 156.8, 129.6, 127.6, 113.7, 101.9, 99.2, 81.9, 70.7, 69.0, 68.9, 62.4, 55.6, 55.4, 55.3, 22.10, 22.06.

Synthesis of Compound **7**. Isobutyl chloroformate (45 µL, 0.34 mmol, 1.2 equiv) was reacted with compound **4** in DCM with DIEA. The reaction was stopped at 1 h. The crude product was purified via column chromatography on silica gel using 0–2% MeOH/DCM (NH_3_) to yield a white solid. Yield: 127 mg (97%). R_f_ = 0.6 in 3% MeOH/DCM. mp = 169.0–170.0 °C. ^1^H NMR (400 MHz, CDCl_3_) *δ* 7.38–7.45 (m, 2H), 6.85–6.93 (m, 2H), 5.51 (s, 1H), 5.03 (s, 1H), 4.73 (d, *J* = 2.81 Hz, 1H), 4.21–4.33 (m, 1H), 3.83–3.98 (m, 4H), 3.70–3.82 (m, 4H), 3.55 (t, *J* = 8.8 Hz, 1H), 3.40 (s, 3H), 2.75 (s, 1H), 1.84–2.05 (m, 1H), 0.94 (d, *J* = 6.7 Hz, 6H). ^13^C NMR (100 MHz, CDCl_3_) *δ* 160.3, 157.3, 129.6, 127.6, 113.7, 101.9, 99.2, 81.9, 71.6, 70.6, 68.9, 62.4, 55.6, 55.4, 55.3, 28.0, 19.0. LC-MS m/z calculated for C_20_H_30_NO_8_ [M+H]^+^ 412.19; found 412.

Synthesis of Compound **8**. Allyl chloroformate (41 µL, 0.38 mmol, 1.2 equiv) was reacted with compound **4** in DCM with DIEA. The reaction was stopped at 3 h. The crude product was purified via column chromatography on silica gel using 0–3% MeOH/DCM (NH_3_) to yield a white solid. Yield: 116 mg (92%). R_f_ = 0.5 in 3% MeOH/DCM. mp = 181.5–182.0 °C. ^1^H NMR (400 MHz, CDCl_3_) *δ* 7.37–7.46 (m, 2H), 6.85–6.93 (m, 2H), 5.86–6.01 (m, 1H), 5.51 (s, 1H), 5.35 (dq, *J*_1_ = 17.2 Hz, *J*_2_ = 1.5 Hz, 1H), 5.26 (dq, *J*_1_ = 10.4 Hz, *J*_2_ = 1.3 Hz, 1H), 5.10 (d, *J* = 7.6 Hz, 1H), 4.73 (d, *J* = 3.38 Hz, 1H), 4.60 (d, *J* = 5.6 Hz, 2H), 4.23–4.29 (m, 1H), 3.83–3.98 (m, 2H), 3.71–3.83 (s, 3H), 3.54 (t, *J* = 8.8 Hz, 1H), 3.40 (s, 3H), 2.68 (s, 1H). ^13^C NMR (100 MHz, CDCl_3_) *δ* 160.3, 156.6, 132.5, 129.6, 127.6, 118.1, 113.7, 102.0, 99.2, 81.9, 70.5, 68.8, 66.1, 62.4, 55.6, 55.4, 55.3. LC-MS m/z calculated for C_19_H_26_NO_8_ [M+H]^+^ 396.15; mass found 396.

Synthesis of Compound **9**. N-butyl chloroformate (84 µL, 0.64 mmol, 2.0 equiv) was reacted with compound **4** in DCM with DIEA. The reaction was stopped at 2 h. The crude product was purified via column chromatography on silica gel using 0–2% MeOH/DCM (NH_3_) to yield a white solid. Yield: 120 mg (92%). R_f_ = 0.4 in 3% MeOH/DCM. mp = 164.5–166.5 °C. ^1^H NMR (400 MHz, CDCl_3_) *δ* 7.38–7.46 (m, 2H), 6.84–6.93 (m, 2H), 5.51 (s, 1H), 4.92–5.15 (m, 1H), 4.73 (d, *J* = 3.04 Hz, 1H), 4.21–4.31 (m, 1H), 4.09 (t, *J* = 6.7 Hz, 2H), 3.82–3.98 (m, 2H), 3.70–3.82 (m, 5H), 3.54 (t, *J* = 8.88 Hz), 3.40 (s, 3H), 2.75 (s, 1H), 1.53–1.65 (m, 2H), 1.33–1.45 (m, 2H), 0.93 (t, 3H, *J* = 7.4 Hz). ^13^C NMR (100 MHz, CDCl_3_) *δ* 160.3, 157.2, 129.6, 127.6, 113.6, 101.9, 99.2, 81.9, 70.6, 68.9, 65.4, 62.4, 55.6, 55.4, 55.3, 31.0, 19.0,13.7. LC-MS m/z calculated for C_20_H_30_NO_8_ [M+H]^+^ 412.19; mass found 412.

Synthesis of Compound **10**. Octyl Chloroformate (77 µL, 0.38 mmol, 1.2 equiv) was reacted with compound **4** in DCM with DIEA. The reaction was stopped at 4 h. The crude product was purified via column chromatography on silica gel using 25% EtOAc/hexanes to 40% EtOAc/hexanes to yield a white solid. Yield: 137 mg (89%). R_f_ = 0.6 in 45% EtOAc/hexanes. mp = 109.0–110.0 °C. ^1^H NMR (400 MHz, CDCl_3_) *δ* 7.36–7.47 (m, 2H), 6.83–6.95 (m, 2H), 5.51 (s, 1H), 5.03 (d, *J* = 6.1 Hz, 1H), 4.73 (d, *J* = 3.0 Hz, 1H), 4.22–4.30 (m, 1H), 4.08 (t, *J* = 6.8 Hz, 2H), 3.83–3.98 (m, 2H), 3.71–3.82 (m, 5H), 3.55 (t, *J* = 8.9 Hz, 1H), 3.40 (s, 3H), 2.75 (s, 1H), 1.56–1.70 (m, 2H), 1.18–1.41 (m, 10H), 0.84–0.93 (m, 3H). ^13^C NMR (100 MHz, CDCl_3_) *δ* 160.3, 157.2, 129.6, 127.6, 113.7, 101.9, 99.2, 81.9, 70.6, 68.9, 65.7, 62.4, 55.6, 55.4, 55.3, 31.8, 29.2 (d), 25.8, 22.6, 14.1. LC-MS m/z calculated for C_24_H_38_NO_8_ [M+H]^+^ 468.25; mass found 468.

Synthesis of Compound **11**. 2,2,2-trichloroethyl chloroformate (47 µL, 0.38 mmol, 1.2 equiv) was reacted with compound **4** in DCM with DIEA. The reaction was stopped at 4 h. The crude product was purified via column chromatography on silica gel using 0–5% MeOH/DCM (NH_3_) to yield a white solid. Yield: 147 mg (94%). R_f_ = 0.6 in 3% MeOH/DCM. mp = 176.5–177.5 °C. ^1^H NMR (400 MHz, CDCl_3_) *δ* 7.37–7.46 (m, 2H), 6.85–6.94 (m, 2H), 5.51 (s, 1H), 5.32 (d, *J* = 8.7 Hz, 1H), 4.67–4.85 (m, 3H), 4.22–4.31 (m, 1H), 3.85–4.00 (m, 2H), 3.71–3.83 (m, 5H), 3.54 (t, *J* = 8.8 Hz, 1H), 3.41 (s, 3H), 2.75 (s, 1H). ^13^C NMR (100 MHz, CDCl_3_) *δ* 160.3, 154.9, 129.5, 127.6, 113.7, 101.9, 99.0, 95.4, 81.8, 74.8, 70.2, 68.8, 62.4, 55.8, 55.4, 55.3. LC-MS m/z calculated for C_18_H_22_Cl_3_NO_8_ [M+H]^+^ 486.04; mass found 486.

Synthesis of Compound **12**. Phenyl Chloroformate (43 µL, 0.34 mmol, 1.05 equiv) was reacted with compound **4** in DCM with DIEA. The reaction was stopped at 2 h. The crude product was purified via column chromatography on silica gel using 0–5% MeOH/DCM (NH_3_) to yield a white solid. Yield: 131 mg (95%). R_f_ = 0.6 in 3% MeOH/DCM. mp = 183.5–185.5 °C. ^1^H NMR (400 MHz, CDCl_3_) *δ* 7.40–7.45 (m, 2H), 7.32–7.39 (m, 2H), 7.13–7.24 (m, 3H), 6.87–6.93 (m, 2H), 5.52 (s, 1H), 5.41 (d, *J* = 7.4 Hz, 1H), 4.83 (d, *J* = 3.2 Hz, 1H), 4.25–4.31 (m, 1H), 3.91–4.04 (m, 2H), 3.71–3.87 (m, 5H), 3.56 (t, *J* = 9.0 Hz, 1H), 3.45 (s, 3H), 2.71 (s, 1H). ^13^C NMR (100 MHz, CDCl_3_) *δ* 160, 155.0, 150.9, 129.5, 127.6, 125.5, 121.5, 113.7, 101.9, 99.0, 81.9, 70.3, 68.8, 62.4, 55.8, 55.4, 55.3. LC-MS m/z calculated for C_22_H_26_NO_8_ [M+H]^+^ 432.16; mass found 432.

Synthesis of Compound **13**. 4-chlorophenyl chloroformate (48 µL, 0.38 mmol, 1.2 equiv) was reacted with compound **4** in DCM with DIEA. The reaction was stopped at 1 h. The crude product was purified via column chromatography on silica gel using 35–90% EtOAc/hexanes to yield a white solid. Yield: 131 mg (95%). R_f_ = 0.6 in 3% MeOH/DCM. mp = 157.5–158.5 °C. ^1^H NMR (400 MHz, CDCl_3_) *δ* 7.30–7.44 (m, 6H), 6.86–6.92 (m, 2H), 5.51 (s, 1H), 5.05–5.23 (s, 3H), 4.73 (d, *J* = 3.2 Hz, 1H), 4.22–4.30 (m, 1H), 3.83–4.00 (m, 2H), 3.69–3.83 (m, 5H), 3.55 (t, *J* = 8.9 Hz), 3.38 (s, 3H), 2.69 (s, 1H). ^13^C NMR (100 MHz, CDCl_3_) *δ* 160.3, 156.6, 136.1, 129.6, 128.6, 128.3, 127.6, 113.7, 101.9, 99.2, 81.9, 70.6, 68.9, 67.3, 62.4, 55.7, 55.4, 55.3. LC-MS m/z calculated for C_22_H_25_ClNO_8_ [M+H]^+^ 466.12; mass found 466.

Synthesis of Compound **14**. *p*-Tolyl chloroformate (49 µL, 0.34 mmol, 1.05 equiv) was reacted with compound **4** in DCM with DIEA. The reaction was stopped at 1 h. The crude product was purified via column chromatography on silica gel using 0–5% MeOH/DCM to yield a white solid. Yield: 133 mg (94%). R_f_ = 0.5 in 3% MeOH/DCM. mp = 157.5–158.5 °C. ^1^H NMR (400 MHz, CDCl_3_) *δ* 7.39–7.46 (m, 2H), 7.09–7.18 (m, 2H), 6.99–7.06 (m, 2H), 6.86–6.94 (m, 2H), 5.52 (s, 1H), 5.38 (d, *J* = 6.8 Hz, 1H), 4.82 (d, *J* = 3.0 Hz, 1H), 4.24–4.31 (m, 1H), 3.87–4.04 (m, 2H), 3.71–3.86 (m, 5H), 3.56 (t, *J* = 8.9 Hz, 1H), 3.44 (s, 3H), 2.69 (s, 1H), 2.33 (s, 3H). ^13^C NMR (100 MHz, CDCl_3_) *δ* 160.3, 155.2, 148.7, 135.1, 129.8, 129.6, 127.6, 121.2, 113.7, 101.9, 99.0, 81.9, 70.4, 68.8, 62.4, 55.8, 55.4, 55.3, 20.8. LC-MS m/z calculated for C_22_H_26_NO_8_ [M+H]^+^ 432.16; mass found 446.

Synthesis of Compound **15** [41,42]. Benzyl chloroformate (49 µL, 0.34 mmol, 1.2 equiv) was reacted with compound **4** in DCM with DIEA. The reaction was stopped at 1 h. The crude product was purified via column chromatography on silica gel using 0–2% MeOH/DCM to yield a white solid. Yield: 132 mg (93%). R_f_ = 0.6 in 3% MeOH/DCM. mp = 180.0–181.5 °C. ^1^H NMR (400 MHz, CDCl_3_) *δ* 7.29–7.45 (m, 7H), 6.85–6.93 (m, 2H), 5.51 (s, 1H), 5.03–5.23 (m, 3H), 4.73 (d, *J* = 3.2 Hz, 1H), 4.21–4.31 (m, 1H), 3.83–4.01 (m, 2H), 3.69–3.82 (m, 5H), 3.55 (t, *J* = 8.9 Hz, 1H), 3.37 (s, 3H), ^13^C NMR (100 MHz, CDCl_3_) *δ* 160.3, 156.8, 136.1, 129.6, 128.6, 128.3, 127.6, 113.7, 101.9, 99.2, 81.9, 70.5, 68.9, 67.3, 62.4, 55.7, 55.4, 55.3. LC-MS m/z calculated for C_23_H_28_NO_8_ [M+H]^+^ 446.17, found 446.

Synthesis of Compound **16**. 4-nitrobenzyl chloroformate (72.4 mg, 0.64 mmol, 1.05 equiv) was reacted with compound **4** in DCM with DIEA. The reaction was stopped at 1 h. The crude product was purified via recrystallization in toluene to yield a white solid. Yield: 137 mg (90%). R_f_ = 0.5 in 3% MeOH/DCM. mp = 214.5–216.5 °C. ^1^H NMR (400 MHz, d_6_-DMSO) *δ* 8.24 (d, *J =* 8.7 Hz, 2H), 7.64 (d, *J =* 8.8 Hz, 2H), 7.45 (d, *J =* 8.5 Hz), 7.34–7.40 (d, 2H), 6.88–6.96 (d, 2H), 5.55 (s, 1H), 5.13–5.26 (m, 3H), 4.68 (d, *J* = 3.53 Hz, 1H), 4.11–4.19 (m, 1H), 3.74–3.80 (s, 3H), 3.52–3.74 (m, 4H), 3.41–3.50 (m, 1H), 3.25–3.56 (m, 3H). ^13^C NMR (100 MHz, CDCl_3_) *δ* 159.5, 155.8, 146.8, 145.0, 130.1, 128.1, 127.7, 123.4, 113.3, 100.8, 98.8, 81.8, 67.9, 67.3, 64.2, 62.4, 56.3, 55.1, 54.8. LC-MS m/z calculated for C_22_H_25_N_2_O_10_ [M+H]^+^ 491.2; mass found 491.

Synthesis of Compound **17**. Fluorenylmethyloxycarbonyl chloride (Fmoc-Cl) (83 mg, 0.34 mmol, 1.05 equiv) was reacted with compound **4** in DCM with DIEA. The reaction was stopped at 2 h. The crude product was purified via recrystallization in toluene to obtain the product as a white solid. Yield: 162 mg (95%). R_f_ = 0.6 in 3% MeOH/DCM. mp = 184.5–185.5 °C. ^1^H NMR (400 MHz, CDCl_3_) *δ* 7.77 (d, *J* = 7.5 Hz, 2H), 7.56–7.66 (m, 2H), 7.37–7.46 (m, 4H), 7.29–7.35 (m, 2H), 6.85–6.91 (m, 2H), 5.51 (s, 1H), 5.11 (d, *J* = 8.0 Hz, 1H), 4.72 (m, 1H), 4.37–4.57 (m, 2H), 4.17–4.35 (m, 2H), 3.65–4.07 (m, 7H), 3.48–3.66 (m, 1H), 3.40 (s, 3H). ^13^C NMR (100 MHz, CDCl_3_) *δ* 160.3, 156.8, 143.9, 141.4, 129.6, 127.7, 127.7, 127.1, 125.1, 125.0, 120.0, 113.7, 101.9, 99.2, 81.9, 70.3, 68.9, 67.0, 62.4, 55.7, 55.4, 55.3, 47.3. LC-MS m/z calculated for C_30_H_32_NO_8_ [M+H]^+^ 534.20; mass found 534.

Synthesis of Compound **18** (Deprotected Octyl). Compound **10** (75 mg, 0.16 mmol, 1 equiv) was dissolved in 80% acetic acid solution (2.5 mL) in a scintillation vial. The reaction mixture was heated to 70 °C for 4 h. Solvent was removed under reduced pressure. The crude product was purified via column chromatography on silica gel using 3–10% MeOH/DCM to yield a white solid. Yield: 54 mg (96%). mp = 110.0–111.0 °C. R_f_ = 0.2 in 5% MeOH/DCM. ^1^H NMR (400 MHz, CDCl_3_) δ 5.23 (d, *J* = 9.1 Hz, 1H), 4.71 (d, *J* = 3.4 Hz, 1H), 3.71–4.20 (m, 7H), 3.52–3.70 (m, 3H), 3.37 (s, 3H), 2.84–3.18 (m, 1H), 1.80–2.00 (m, 2H), 1.52–1.70 (m, 2H), 1.18–1.41 (m, 10H), 0.80–0.94 (m, 3H). LC-MS m/z calculated for C_16_H_32_NO_7_ 350.2 [M+H]^+^ mass found 350.

Synthesis of Compound **19** (Deprotected Benzo). Compound **12** (45 mg, 0.16 mmol, 1 equiv) was dissolved in 80% acetic acid solution (1.25 mL) in a scintillation vial. The reaction mixture was heated to 70 °C for 4 h. Solvent was removed under reduced pressure. The crude product was purified via column chromatography on silica gel using 5–10% MeOH/DCM to yield a white solid. Yield: 21 mg (92%). mp = 131.0–133.0 °C. R_f_ = 0.1 in 5% MeOH/DCM. ^1^H NMR (400 MHz, D_2_O) δ 7.54 (t, *J* = 7.6 Hz, 2H), 7.35–7.46 (m, 1H), 7.25 (d, *J* = 7.8 Hz, 2H), 4.90–5.00 (m, 1H), 3.73–4.01 (m, 6H), 3.45–3.63 (m, 5H).

Synthesis of Compound **20** [43]. (Deprotected Benzyl). Compound **15** (35 mg, 0.16 mmol, 1 equiv) was dissolved in 80% acetic acid solution (1.25 mL) in a scintillation vial. The reaction mixture was heated to 70 °C for 4 h. Solvent was removed under reduced pressure. The crude product was purified via column chromatography on silica gel using 5–10% MeOH/DCM to yield a white solid. Yield: 30 mg (93%). mp = 154.5–155.5 °C. R_f_ = 0.2 in 5% MeOH/DCM. ^1^H NMR (400 MHz, CDCl_3_) δ 7.29–7.43 (m, 5H), 5.03–5.27 (m, 3H), 4.71 (d, *J* = 3.6 Hz), 3.74–3.93 (m, 3H), 3.53–3.73 (m, 3H), 3.37 (s, 3H).

Synthesis of compound **21** [44]. (Deprotected Fmoc Derivative). Compound **17** (96 mg, 0.18 mmol, 1 equiv) was dissolved in 75% acetic acid solution (1.5 mL) in a scintillation vial. The reaction mixture was heated to 30 °C for 20.5 h. The solvent was removed under reduced pressure. The crude product was purified via column chromatography on silica gel using 1–15% MeOH/DCM to yield a white solid, 52 mg (70%, unoptimized yield). mp = 165.5–166.5 °C. R_f_ = 0.03 in 3% MeOH/DCM. ^1^H NMR (400 MHz, d_6_-DMSO) δ 7.89 (d, *J* = 7.6 Hz, 2H), 7.71–7.79 (m, 2H), 7.42 (t, *J* = 7.1 Hz, 2H), 7.33 (t, *J* = 7.4 Hz, 2H), 7.18 (d, *J* = 8.0 Hz, 1H), 4.96 (d, *J* = 5.5 Hz, 1H), 4.72 (d, *J* = 5.6 Hz, 1H), 4.57 (d, *J* = 3.4, 1H), 4.49 (t, *J* = 5.9 Hz, 1H), 4.16–4.34 (m, 3H), 3.58–3.72 (m, 1H), 3.43–3.54 (m, 2H), 3.35–3.43 (m, 1H), 3.26 (s, 3H), 3.09–3.19 (m, 1H).

## Figures and Tables

**Table 1:** Gelation properties of the compound series **III** and deprotected carbamate **IV**.

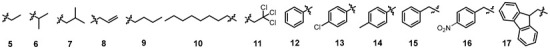												
	Toluene	EtOH	i-PrOH	n-PrOH	n-BuOH	EG	Glycerol	EtOH:H_2_O 1:1	EtOH:H_2_O 1:2	DMSO:H_2_O 1:1	DMSO:H_2_O 1:2	H_2_O
**5**	G_O_ 4.0	G_O_ 10.0	P	G_O_ 10.0	G_O_ 10.0	G_O_ 20.0	G_O_ 20.0	G_O_ 5.0	G_O_ 5.0	G_O_ 6.7	G_O_ 5.0	G_O_ 5.0
**6**	G_C_ 6.7	G_O_ 6.7	G_O_ 6.7	G_O_ 10.0	G_O_ 10.0	G_T_ 6.7	G_C_ 6.7	G_O_ 3.3	G_O_ 6.7	G_O_ 6.7	G_O_ 20	I
**7**	S	G_O_ 6.7	G_O_ 10.0	S	S	G_O_ 6.7	G_O_ 20.0	G_O_ 2.2	G_O_ 1.8	G_O_ 4.0	G_O_ 3.3	G_O_ 1.5
**8**	P	G_O_ 6.7	P	G_O_ 10.0	S	G_O_ 10.0	S	G_O_ 6.7	G_O_ 5.0	G_O_ 6.7	P	I
**9**	P	P	P	P	P	P	S	G_O_ 5.0	G_O_ 2.2	G_O_ 4.0	G_O_ 5.0	G_T_ 0.9
**10**	S	S	S	S	S	S	G_C_ 10.0	P	P	P	P	I
**11**	P	G_O_ 10.0	G_O_ 20.0	G_O_ 20.0	G_O_ 20.0	S	UG	G_O_ 6.7	P	P	P	I
**12**	P	G_O_ 4.0	G_O_ 5.0	G_O_ 10.0	G_O_ 10.0	G_O_ 20.0	G_O_ 10.0	G_O_ 5.0	G_O_ 5.0	G_O_ 5.0	G_O_ 5.0	P
**13**	S	P	P	S	S	P	S	I	I	P	P	I
**14**	S	S	S	S	S	G_O_ 20.0	S	P	P	P	P	I
**15**	G_O_ 5.0	P	G_O_ 10.0	G_O_ 10.0	G_O_ 20.0	G_O_ 10.0	G_O_ 10.0	G_O_ 2.2	P	G_T_ 2.2	G_O_ 4.0	I
**16**	P	S	S	S	S	S	S	I	I	S	P	I
**17**	G_T_ 10.0	G_T_ 2.8	G_T_ 5.0	G_T_ 4.0	G_T_ 5.0	G_T_ 5.0	G_T_ 20.0	G_T_ 0.8	I	G_T_ 1.7	G_T_ 1.0	I
**18**	G_T_ 2.9	S	S	S	S	S	S	P	G_O_ 10.0	G_O_ 10.0	G_O_ 10.0	G_O_10.0
**19**	G_O_ 6.7	S	S	S	S	S	S	S	S	S	S	P
**20**	Gc2.9	P	S	S	S	S	S	P	P	S	S	I
**21**	G_O_ 6.7	G_O_ 10.0	G_O_ 10.0	G_O_ 20.0	G_O_ 10.0	G_O_ 10.0	G_C_ 5.0	G_O_ 5.0	G_C_ 1.0	G_O_ 10.0	G_T_ 4.0	G_T_ 0.9

All compounds were tested starting from 20 mg/mL. G, stable gel at room temperature, the numbers are MGC in mg/mL; P, precipitation; S, soluble; I, insoluble; O, opaque; T, translucent; C, clear, all ratios in mixed solvents are in volume ratio.

## Data Availability

Not applicable.

## Supplementary Materials

The following supporting information can be downloaded at: https://www.mdpi.com/article/10.3390/gels9060445/s1, Figures S1–S19, Part I: ^1^H and ^13^C NMR spectra of compounds **5**–**17** and ^1^H NMR spectra for compounds **18**–**21**. Figures S20–S27, Part II: Additional Experimental for Drug delivery Studies. Figure S20. (a) UV–Vis spectra of chloroquine diphosphate dilution study, (b) Standard curve of chloroquine diphosphate (CQ)*;* Figure S21. (a) Gel photographs of compound **9** with chloroquine at different time points after the pH 7 buffer was removed, (b) Percent release profiles of chloroquine diphosphate to a pH 7.0; Figure S22. Gel photographs of compound **9** with chloroquine at different time points after the pH 3 buffer was removed, (b) Percent release profiles of chloroquine diphosphate to a pH 3.0. Figure S23. (a). UV–Vis spectra of naproxen sodium release study (Trial 2), (b) Percent release of naproxen sodium over time (Trial 2). Figure S24 Concentration (mM) of naproxen sodium released over time (Trial 2). Figure S25 The naproxen standard calibration from the UV–Vis absorption. Figure S26. Compound **10** and pH 1 and pH 4 aqueous solution mixture, before and after heating, Figure S27. Optical micrograph of the sample from heating compound **10** in pH 1 solution.
