# Supplementary material for: Para-Methoxybenzylidene Acetal-Protected D-Glucosamine Derivatives as pH-Responsive Gelators and Their Applications for Drug Delivery
Source: Gels. 2023 May 27;9(6):445. doi: 10.3390/gels9060445 (PMC10298149; doi:10.3390/gels9060445)
Supplement: Supplementary file 1 [file gels-09-00445-s001.zip › gels-2403638-supplementary.pdf]

gels

Supplementary Information

Para-Methoxybenzylidene Acetal-Protected D-Glucosamine Derivatives as pH Responsive Gelators and their Applications for Drug Delivery

Jonathan Bietsch, Logan Baker, Anna Duffney, Alice Mao, Mary Olson, Cheandri Ackermann and Guijun Wang *

Department of Chemistry and Biochemistry, Old Dominion University, Norfolk, VA 23529, USA; jbietsch@odu.edu (J.B.); lbake004@odu.edu (L.B.); aduff003@odu.edu (A.D.); ali.mao6464@gmail.com (A.M.); molso006@odu.edu (M.O.); cacke003@odu.edu (C.A.)

* Correspondence: g1wang@odu.edu; Tel.: +1-757-683-3781

Part I. NMR spectra for compounds synthesized

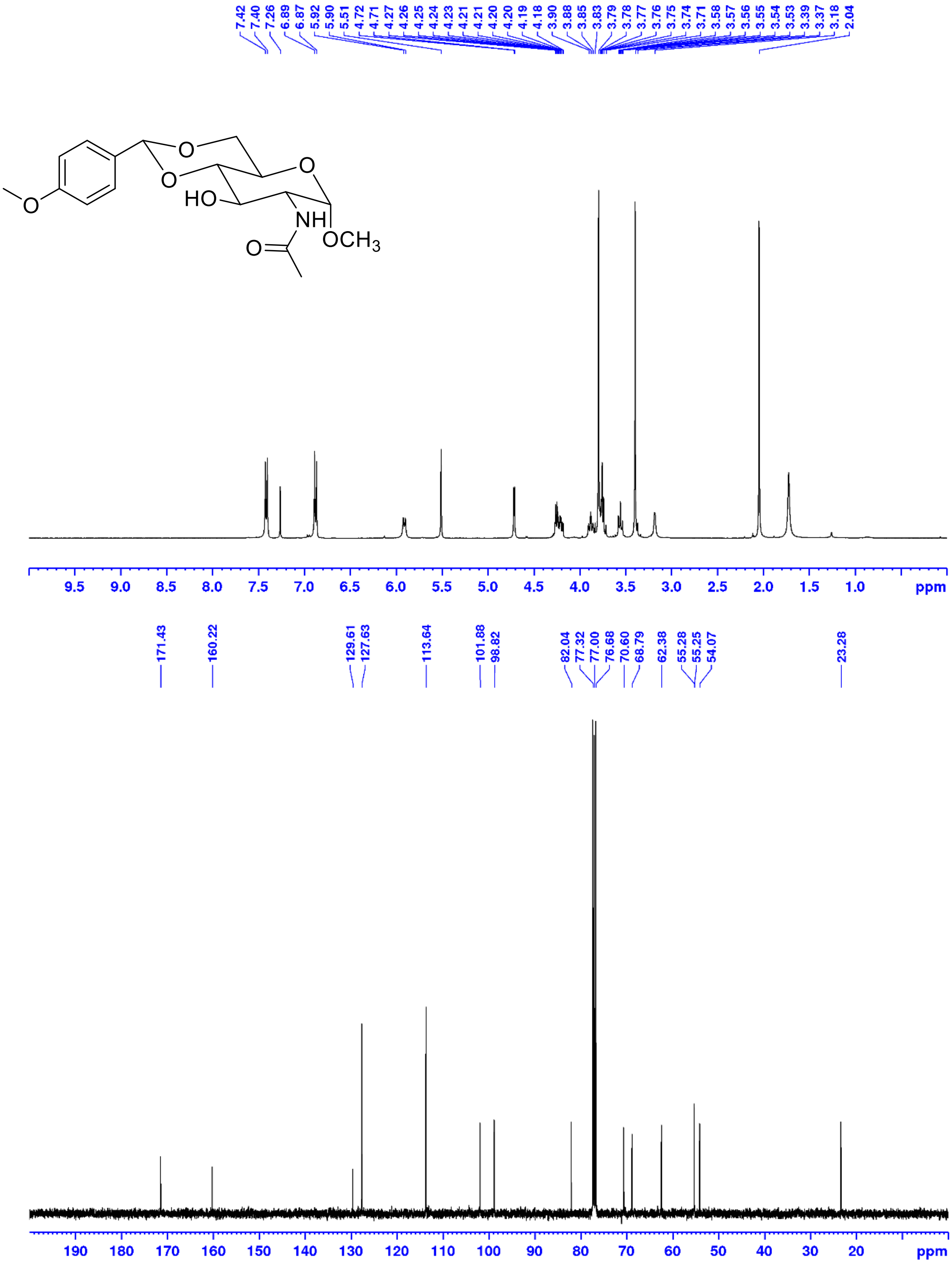

Figure S1. ^{1}H NMR and ^{13}C NMR spectra of compound **3** in $CDCl_3$.

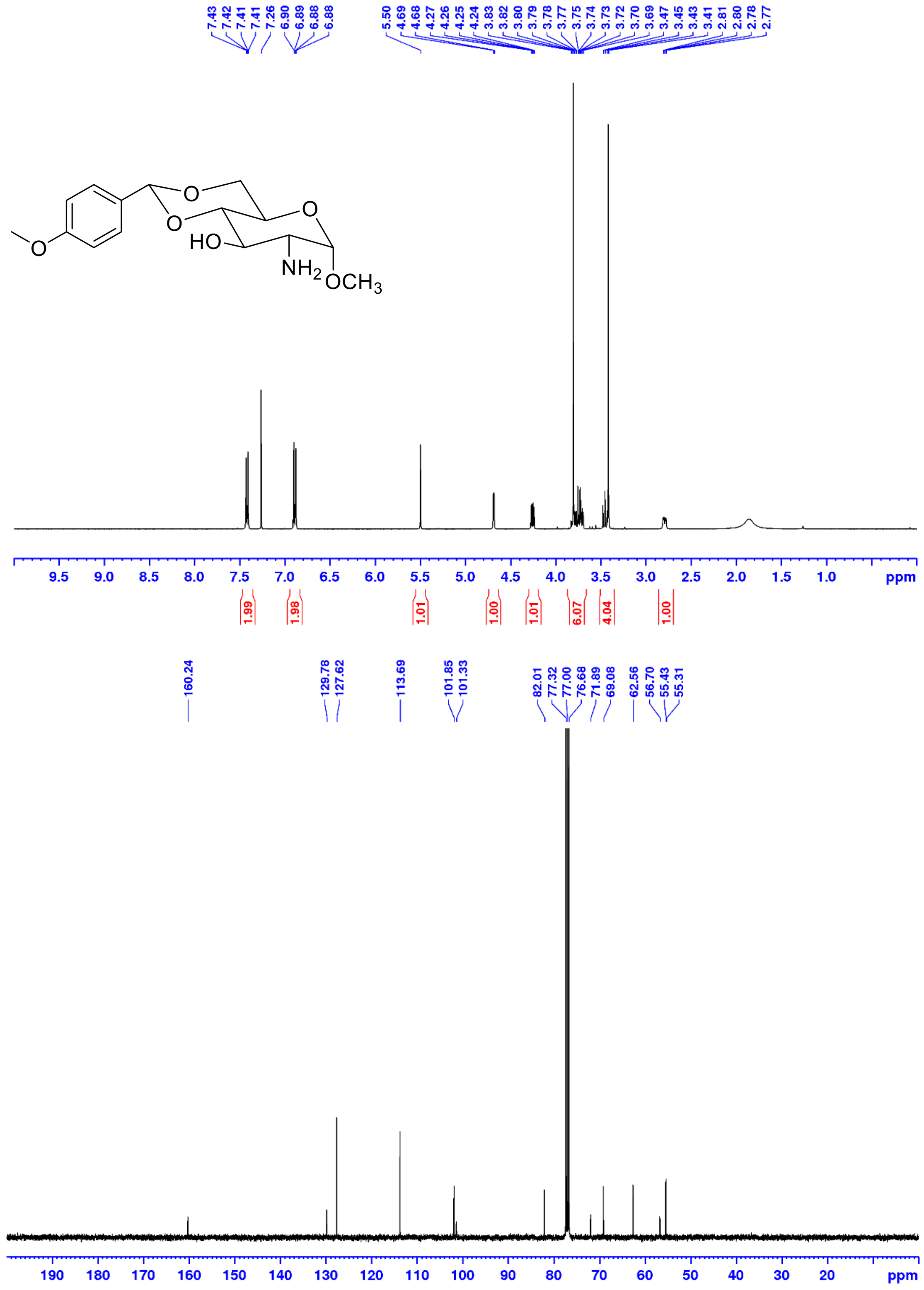

Figure S2. ^{1}H NMR and ^{13}C NMR spectra of compound **4** in $CDCl_3$.

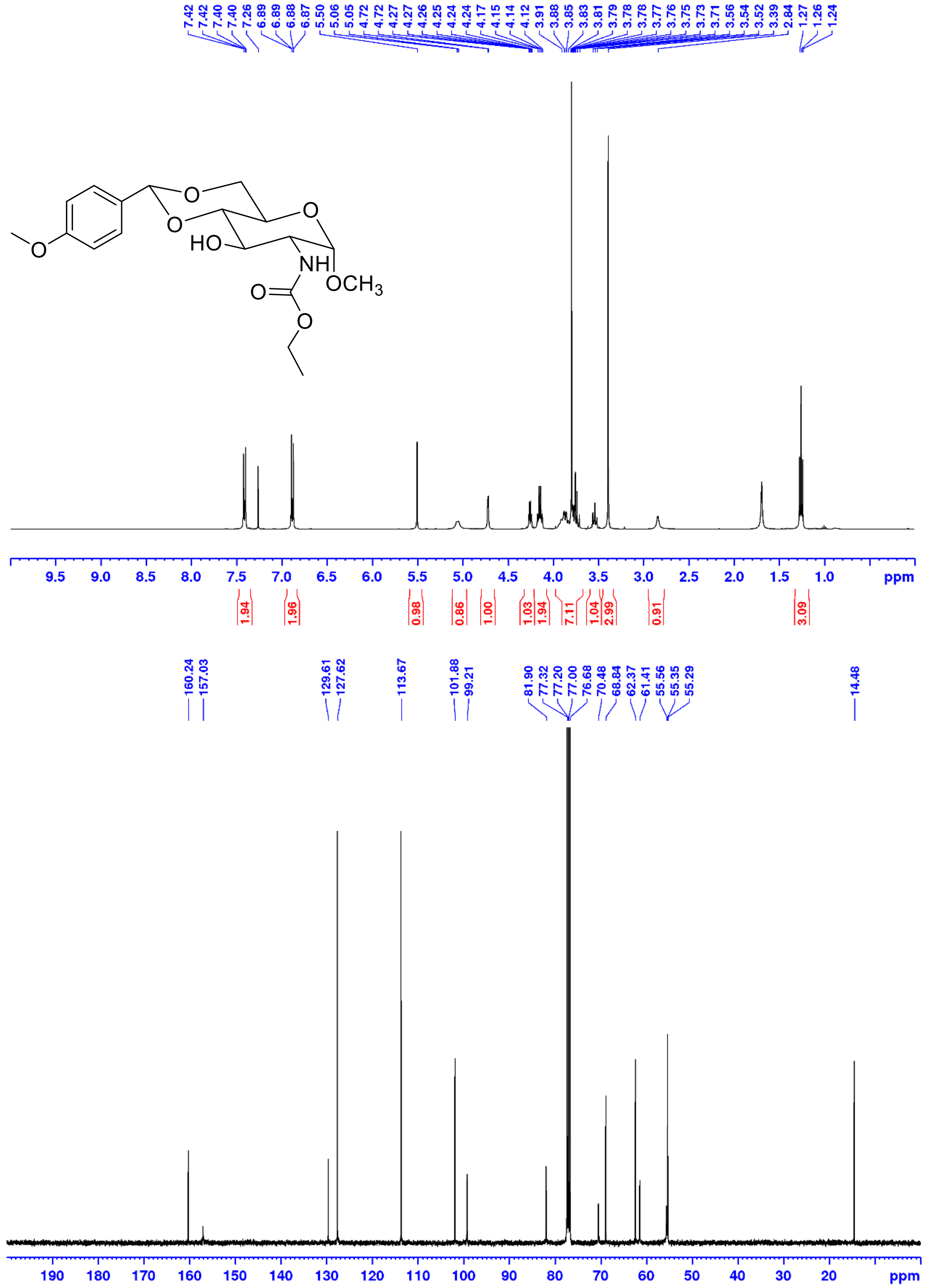

Figure S3. ^1H NMR and ^{13}C NMR spectra of compound **5** in $CDCl_3$.

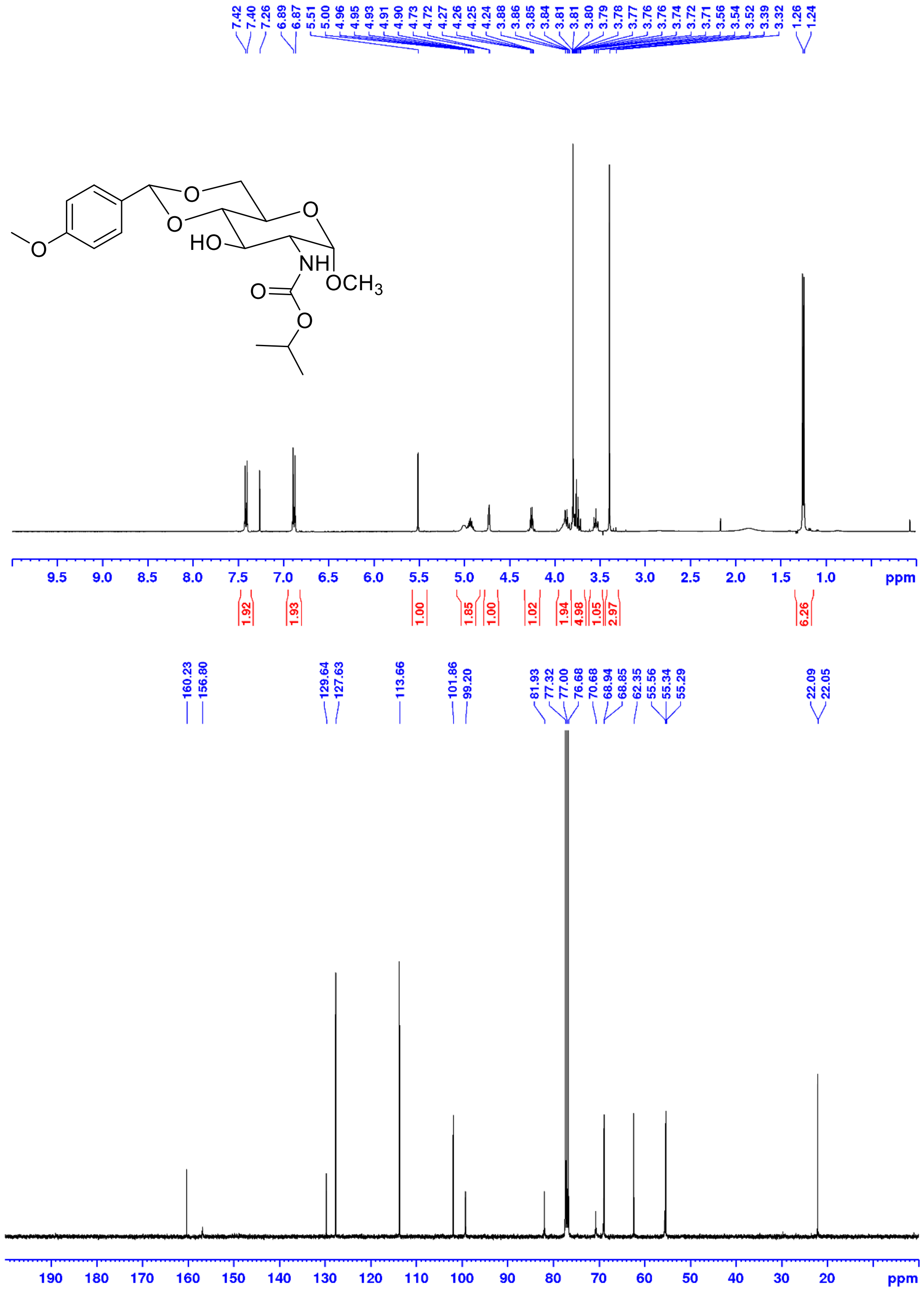

Figure S4. ^{1}H NMR and ^{13}C NMR spectra of compound **6** in $CDCl_3$.

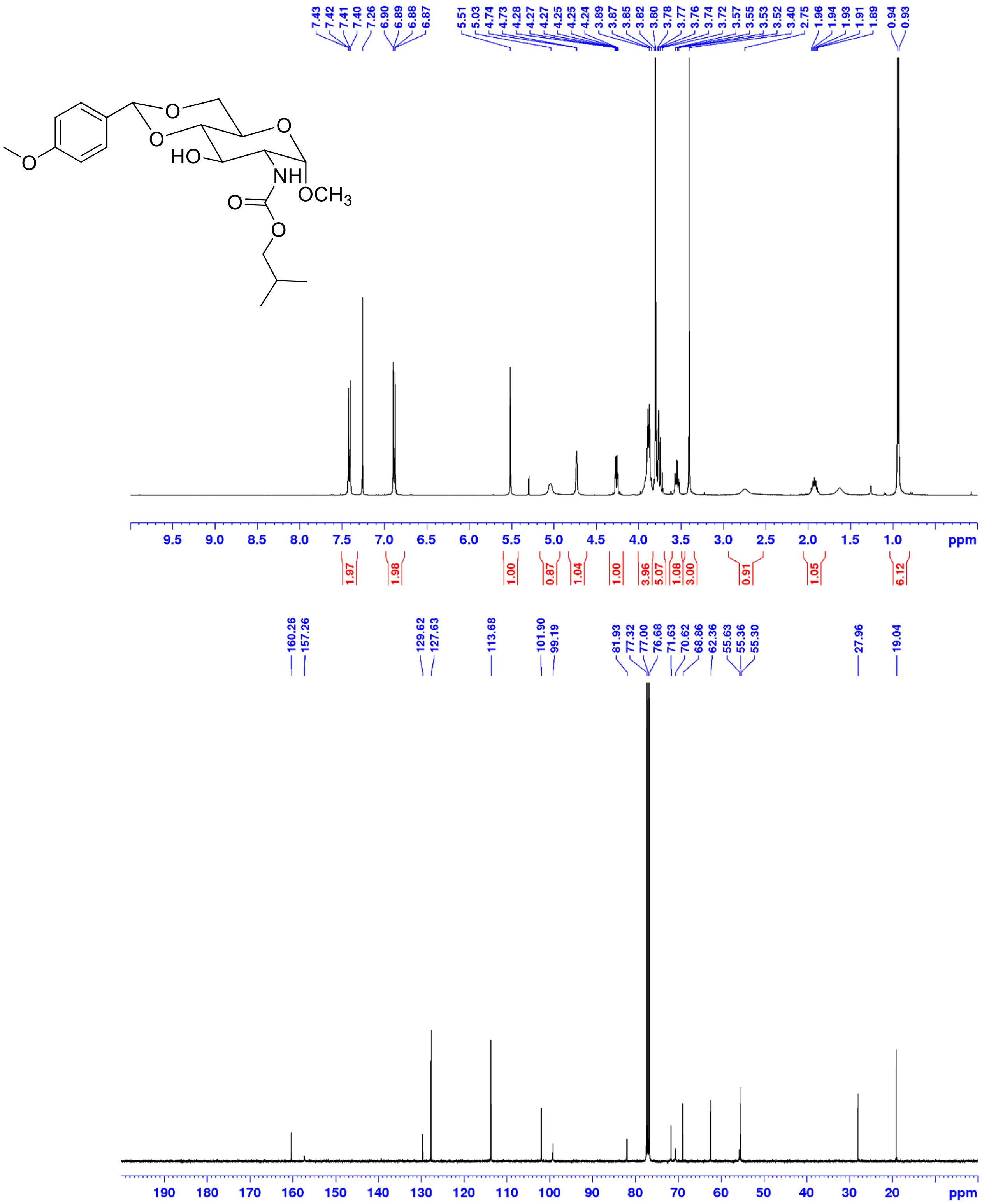

Figure S5. ^{1}H NMR and ^{13}C NMR spectra of compound **7** in $CDCl_3$.

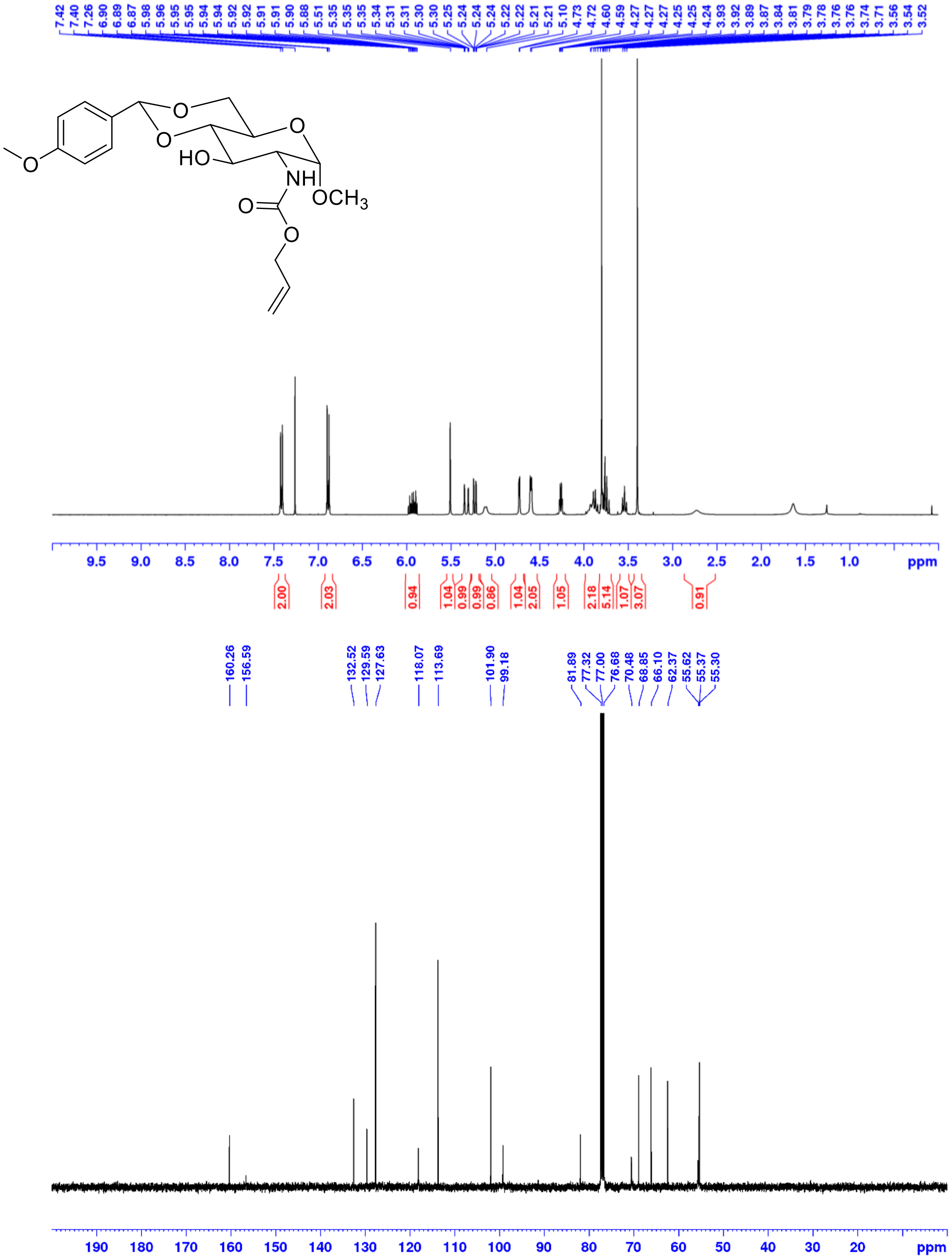

Figure S6. ^{1}H NMR and ^{13}C NMR spectra of compound **8** in $CDCl_3$.

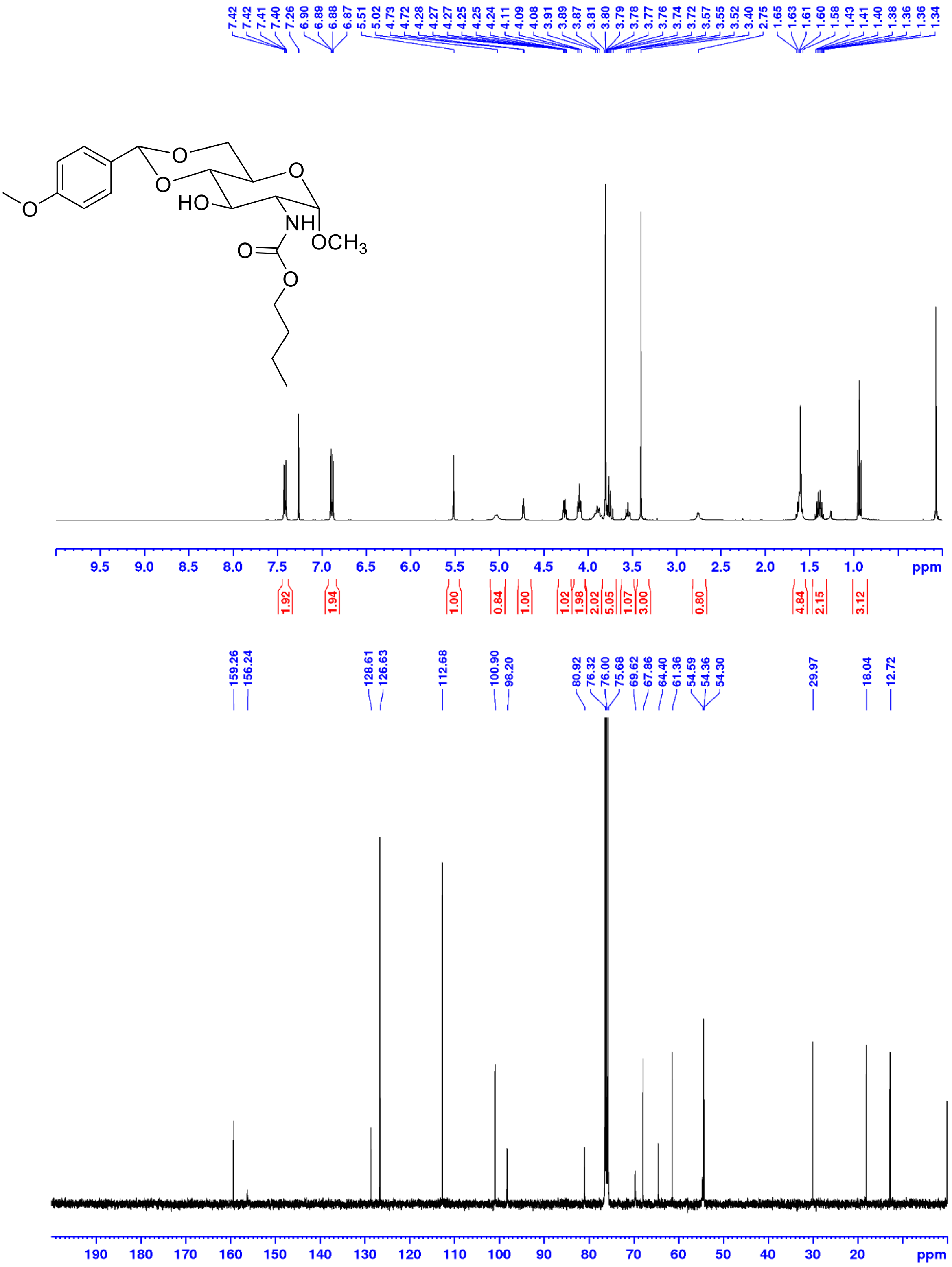

Figure S7. ^{1}H NMR and ^{13}C NMR spectra of compound **9** in $CDCl_3$.

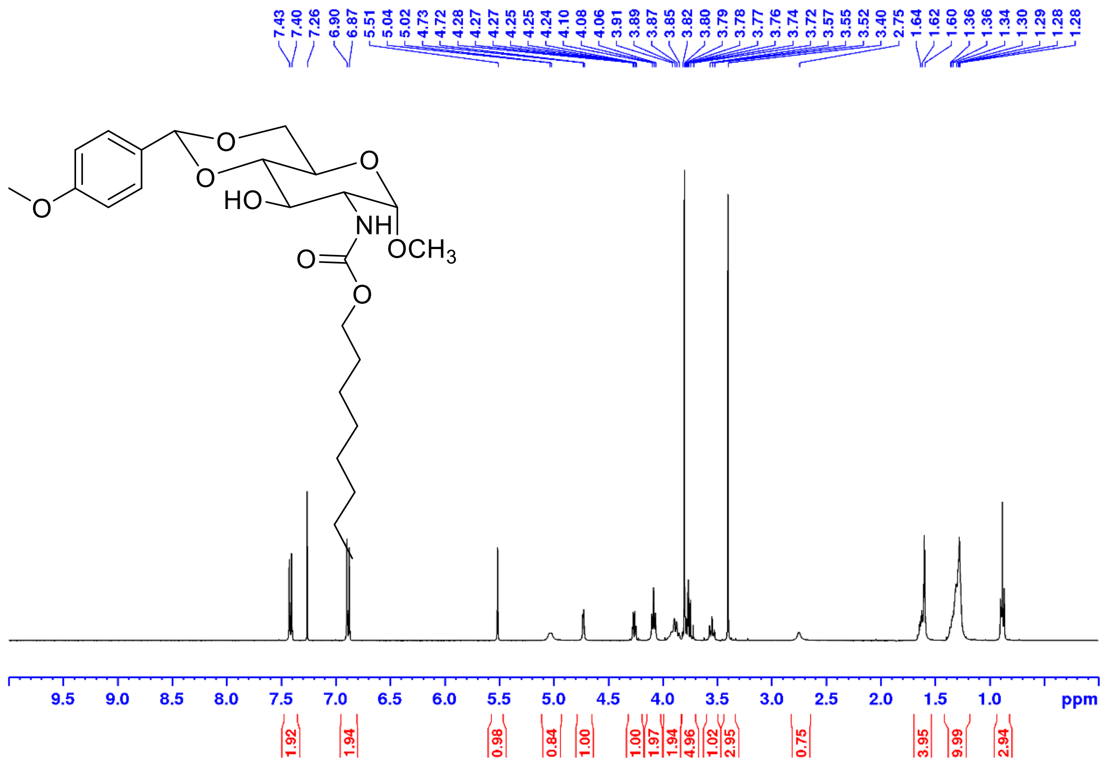

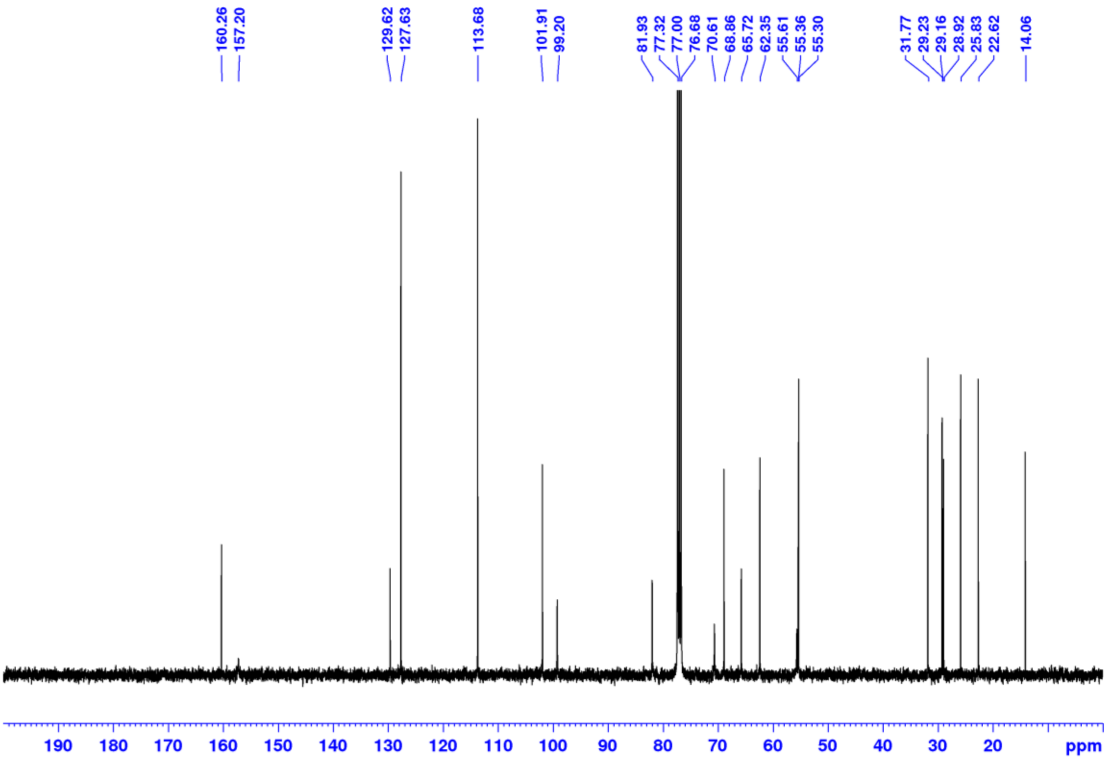

Figure S8. ^{1}H NMR and ^{13}C NMR spectra of compound **10** in $CDCl_3$.

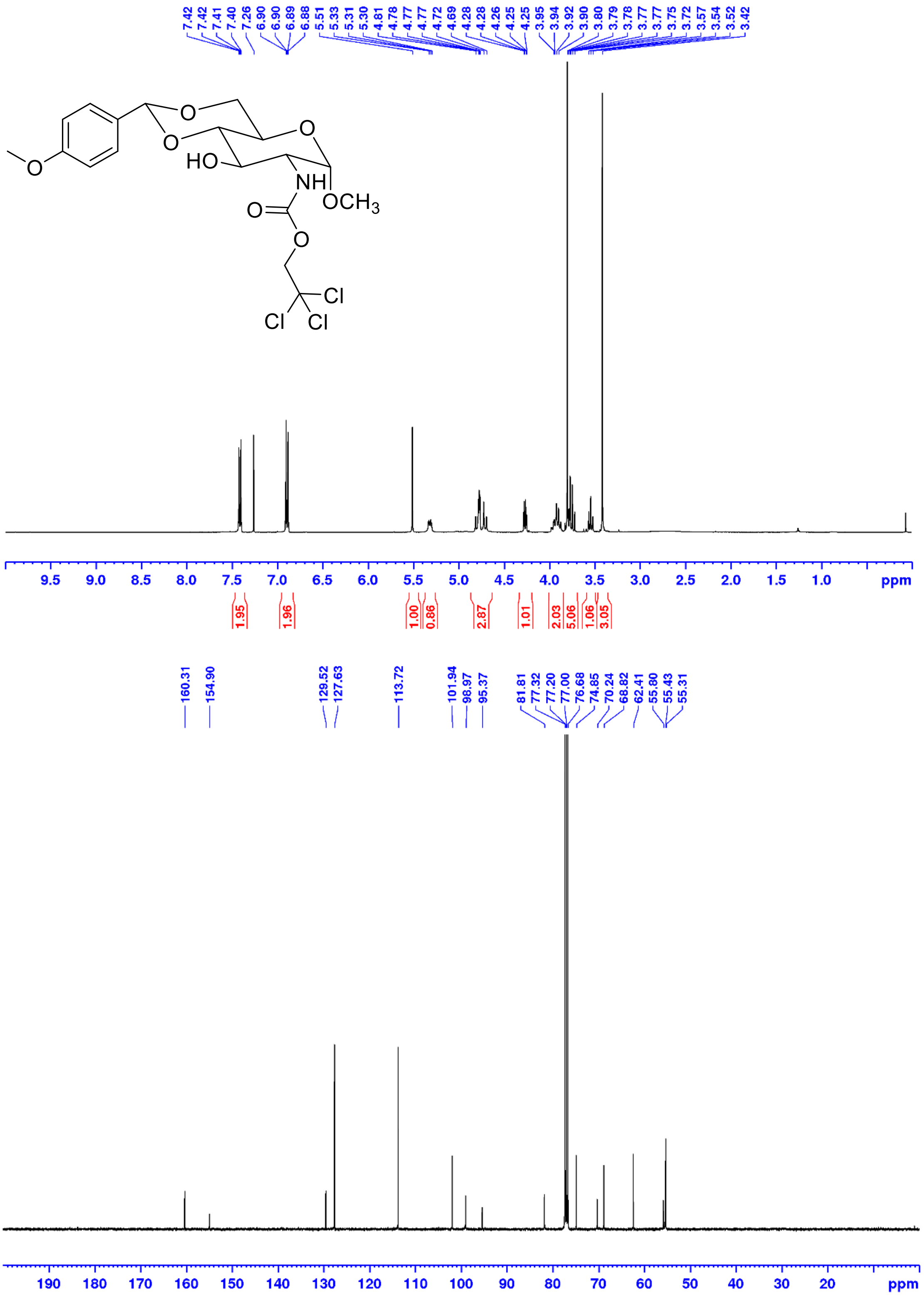

Figure S9. ^{1}H NMR and ^{13}C NMR spectra of compound **11** in $CDCl_3$.

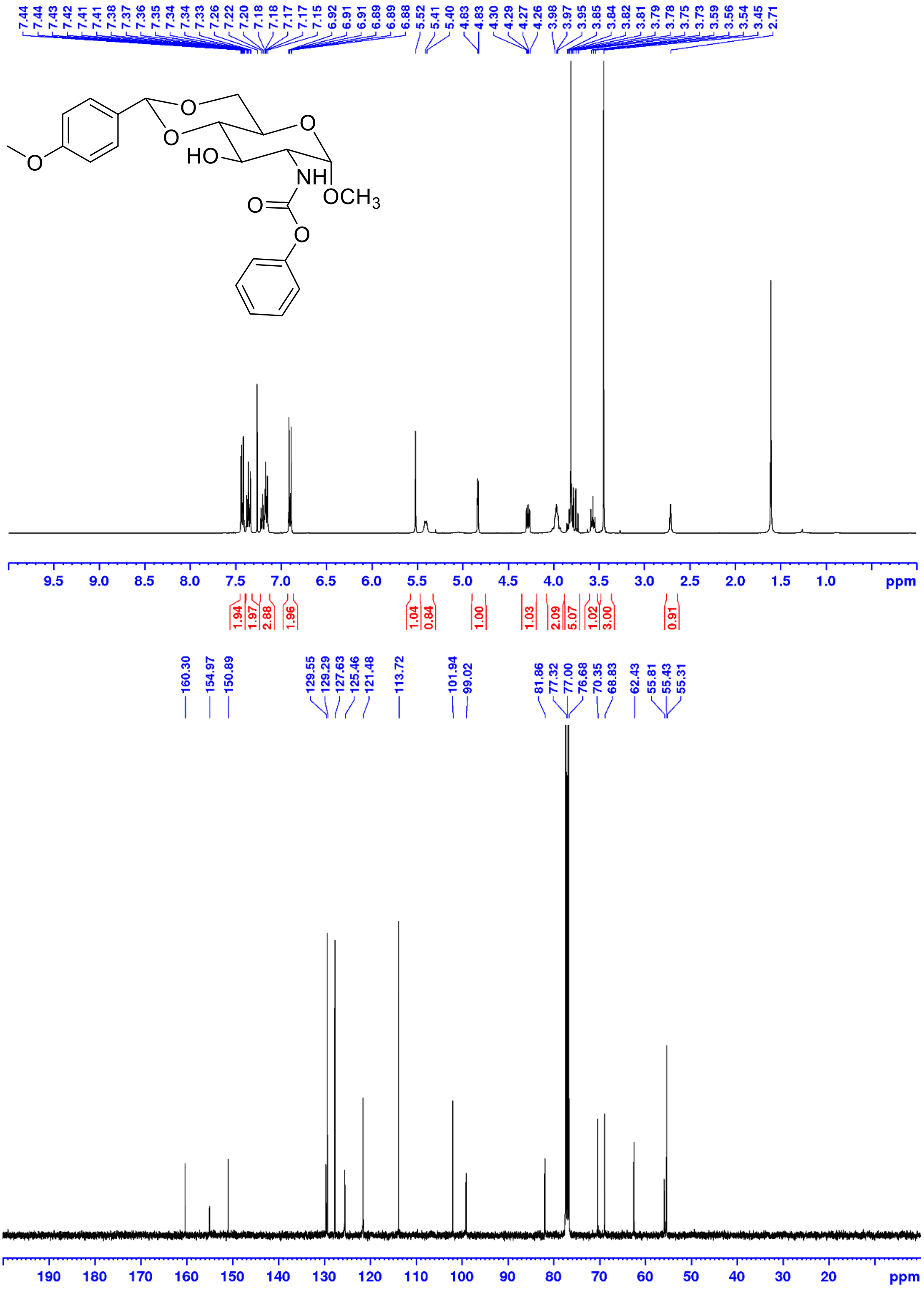

Figure S10. ^{1}H NMR and ^{13}C NMR spectra of compound **12** in $CDCl_3$.

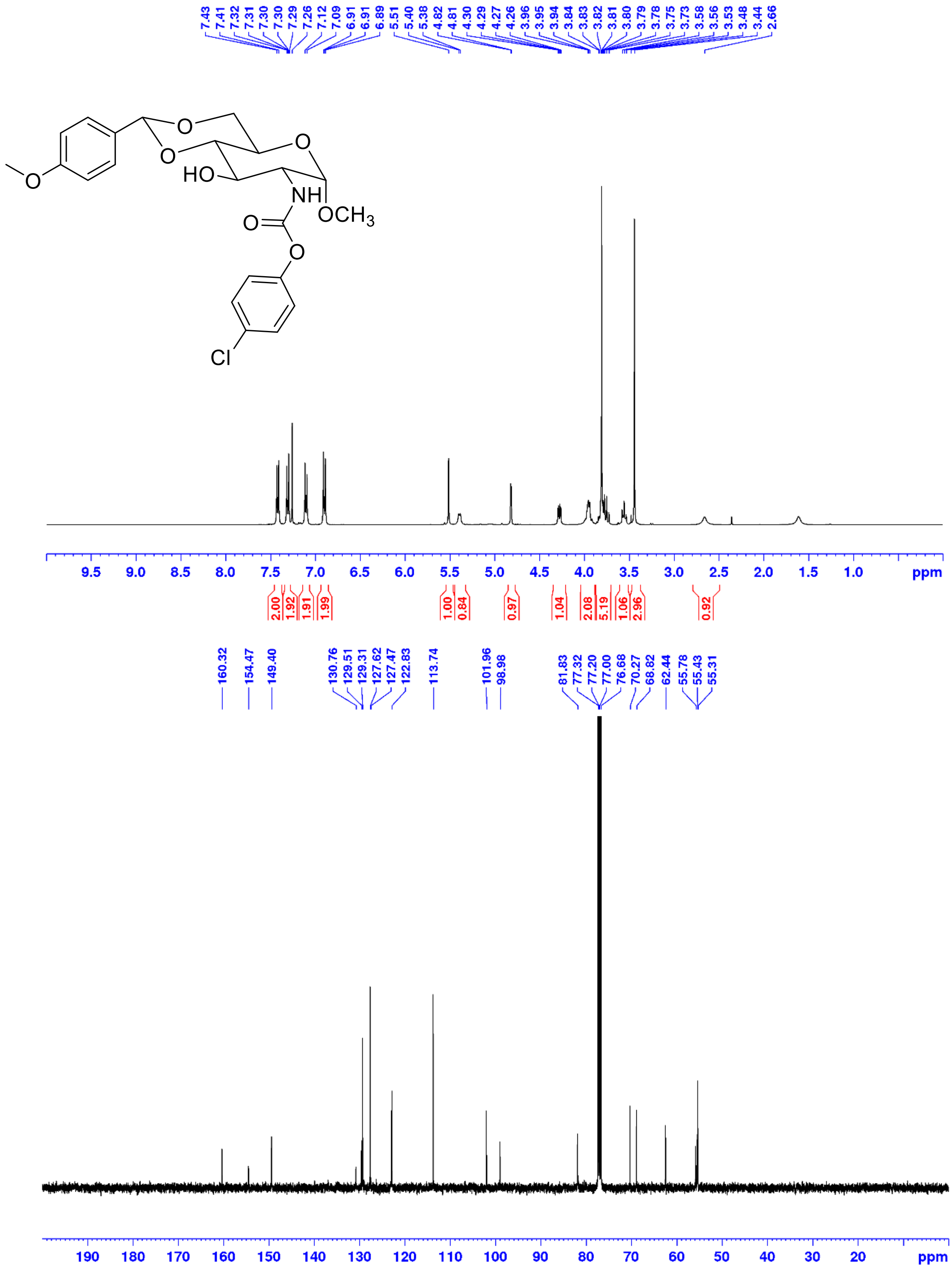

Figure S11. ^{1}H NMR and ^{13}C NMR spectra of compound **13** in $CDCl_3$.

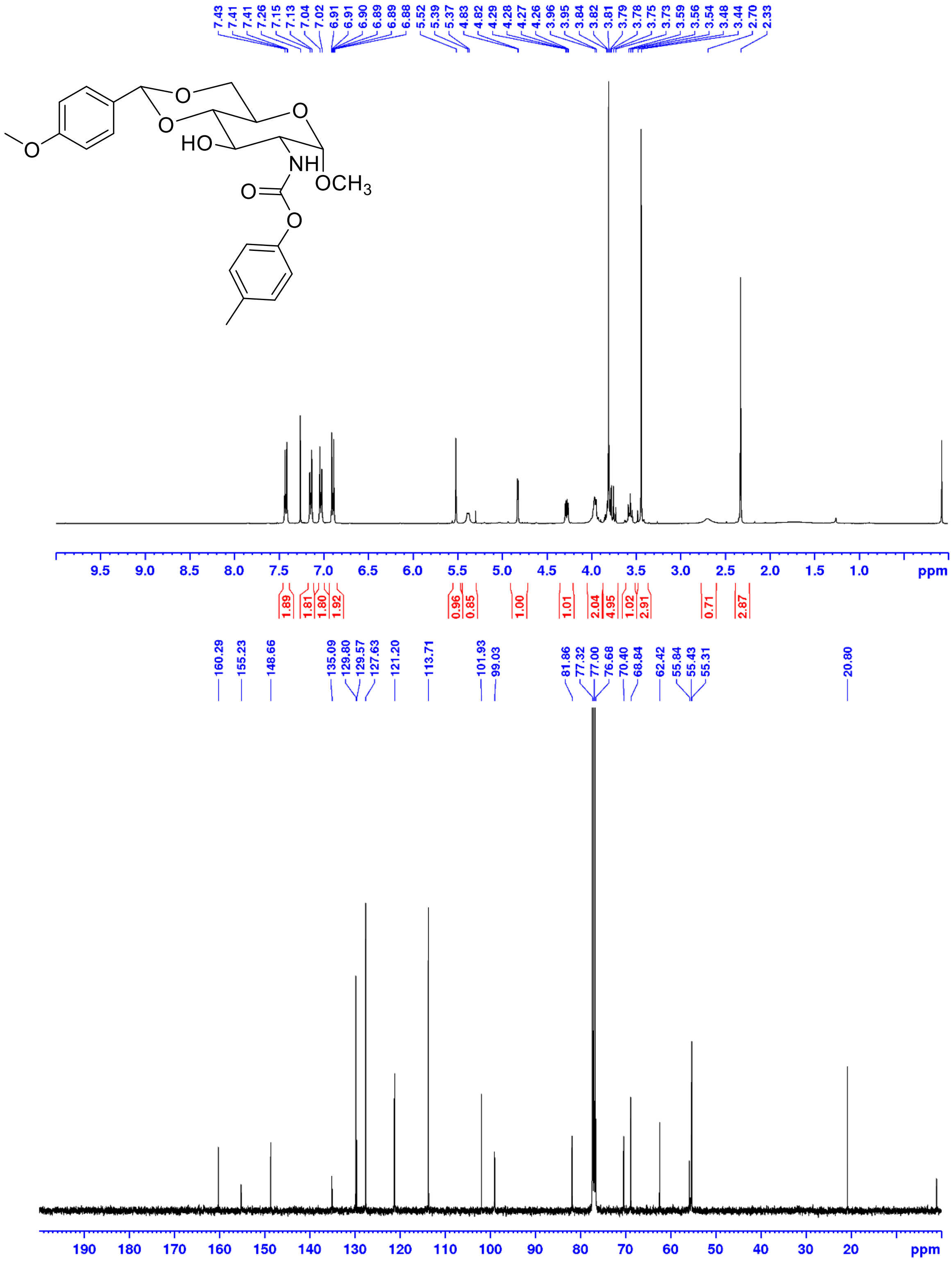

Figure S12. ^{1}H NMR and ^{13}C NMR spectra of compound **14** in $CDCl_3$.

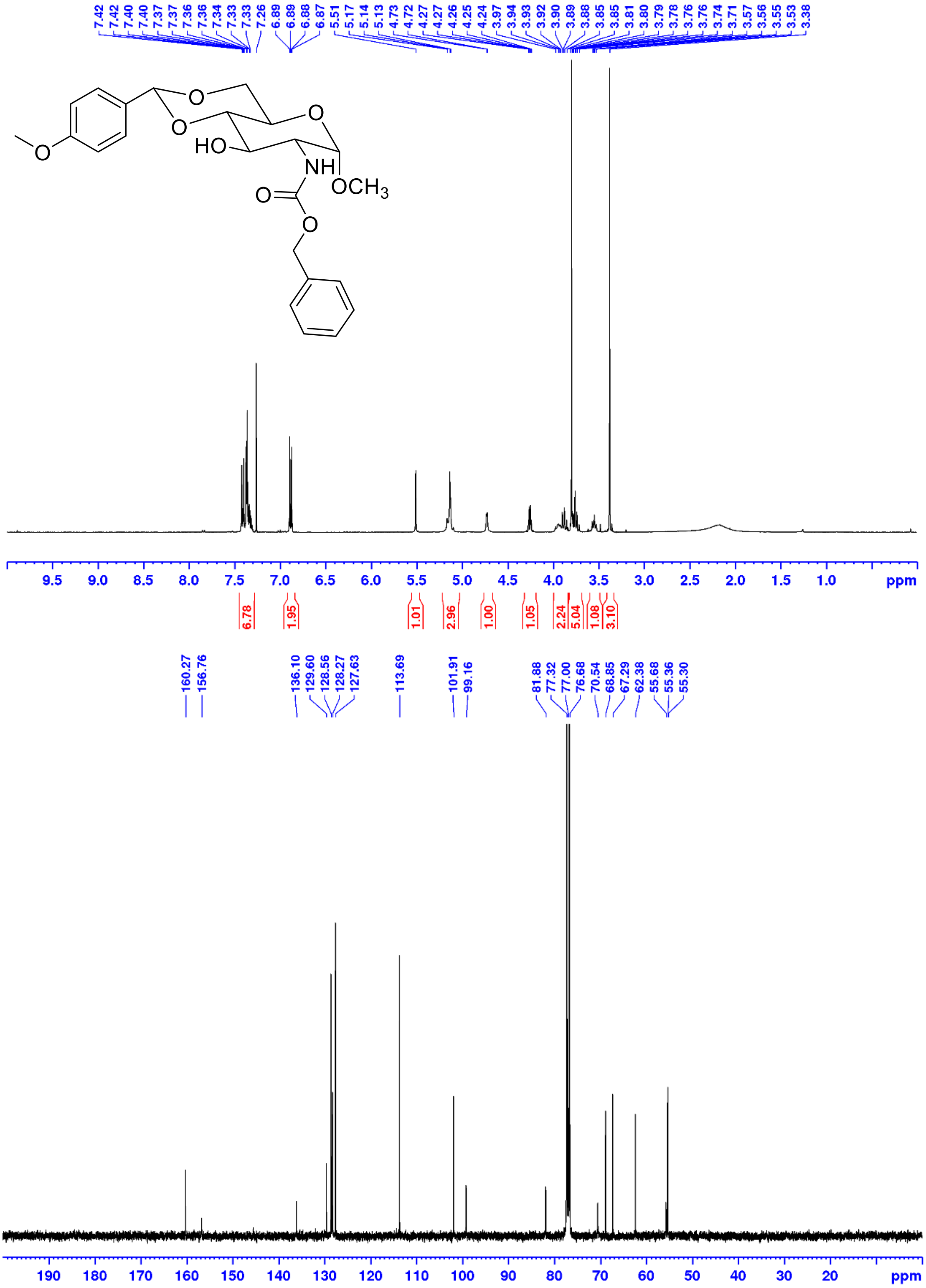

Figure S13. ^{1}H NMR and ^{13}C NMR spectra of compound **15** in $CDCl_3$.

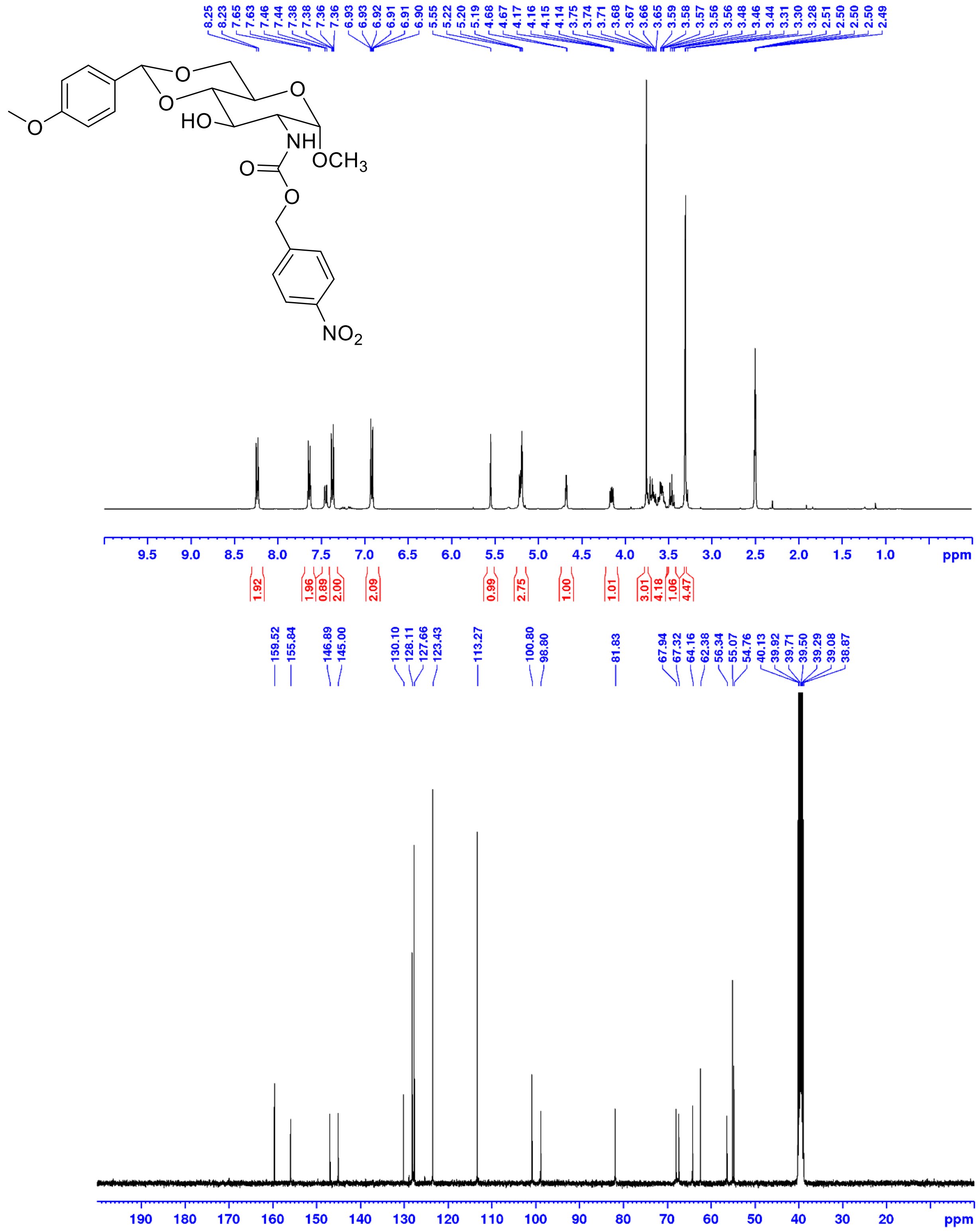

Figure S14. ^{1}H NMR and ^{13}C NMR spectra of compound **16** in d$_6$-DMSO.

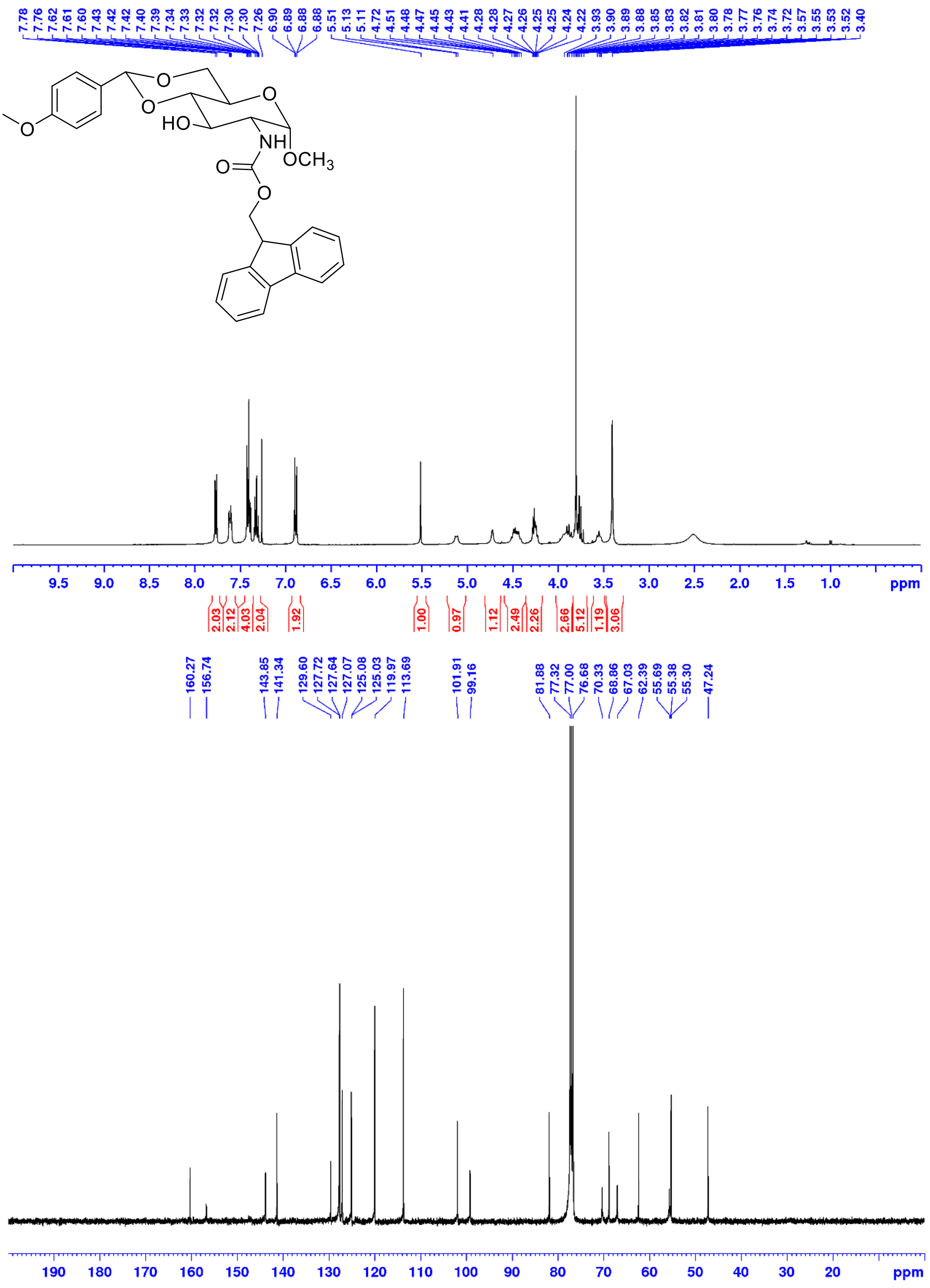

Figure S15. ^{1}H NMR and ^{13}C NMR spectra of compound **17** in $CDCl_3$.

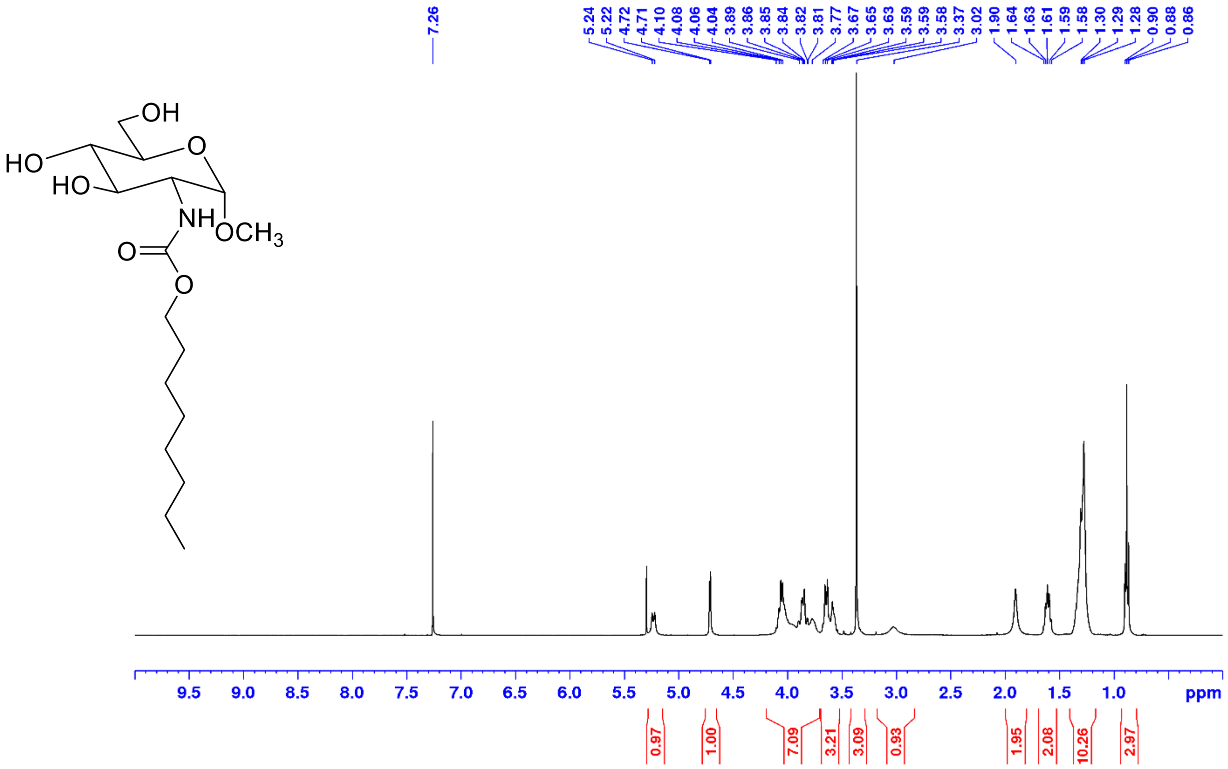

Figure S16. ^{1}H NMR spectrum of compound **18** in $CDCl_3$.

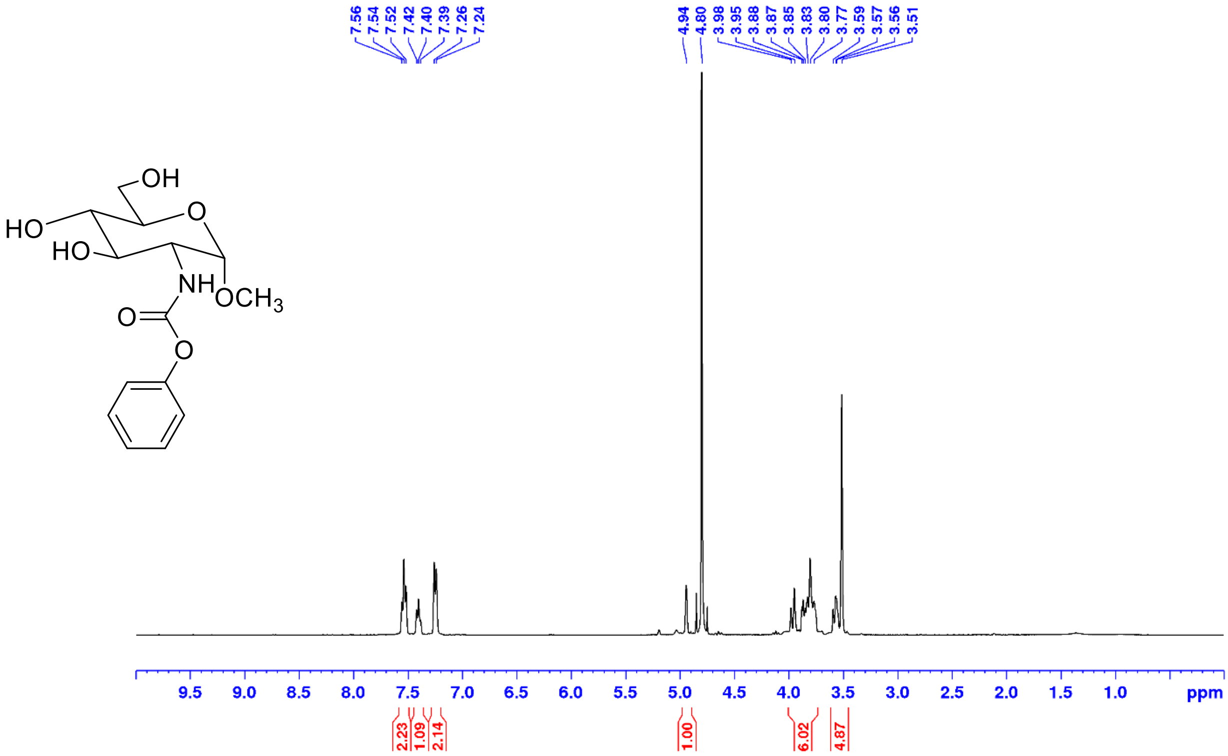

Figure S17. ^{1}H NMR spectrum of compound **19** in D_2O.

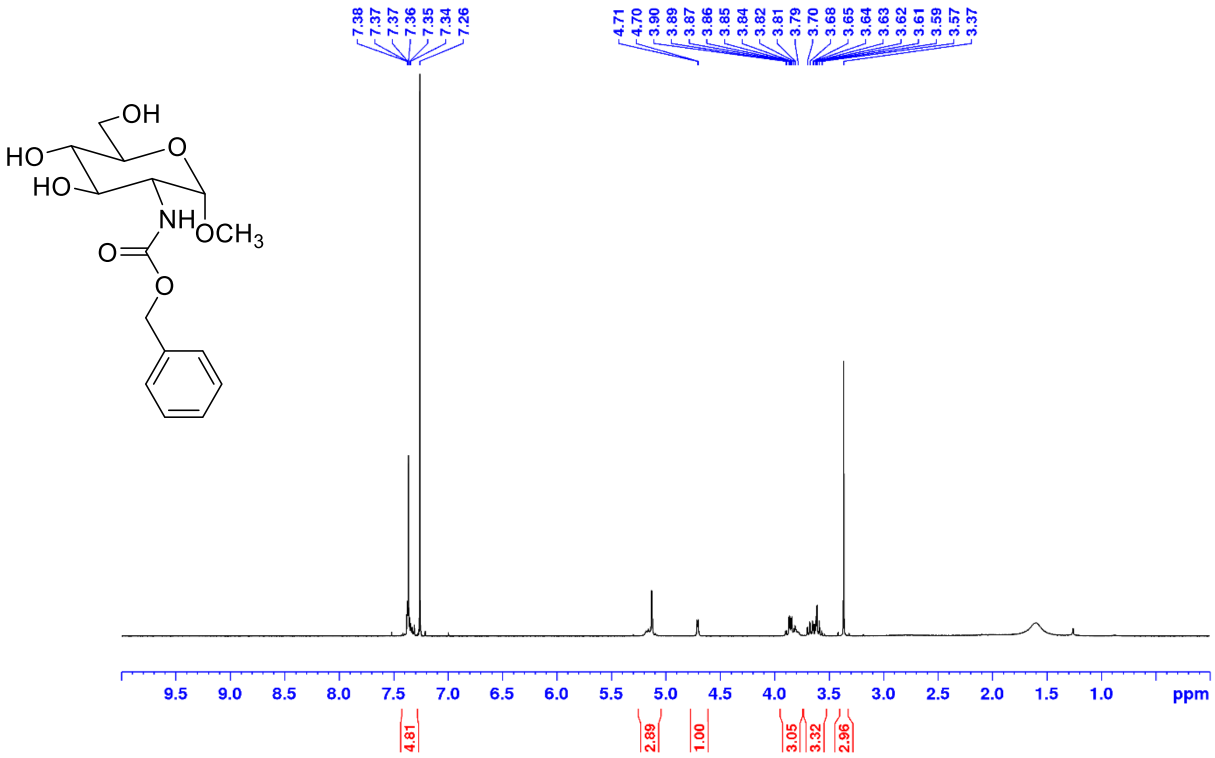

Figure S18. ¹H NMR spectrum of compound **20** in $CDCl_3$.

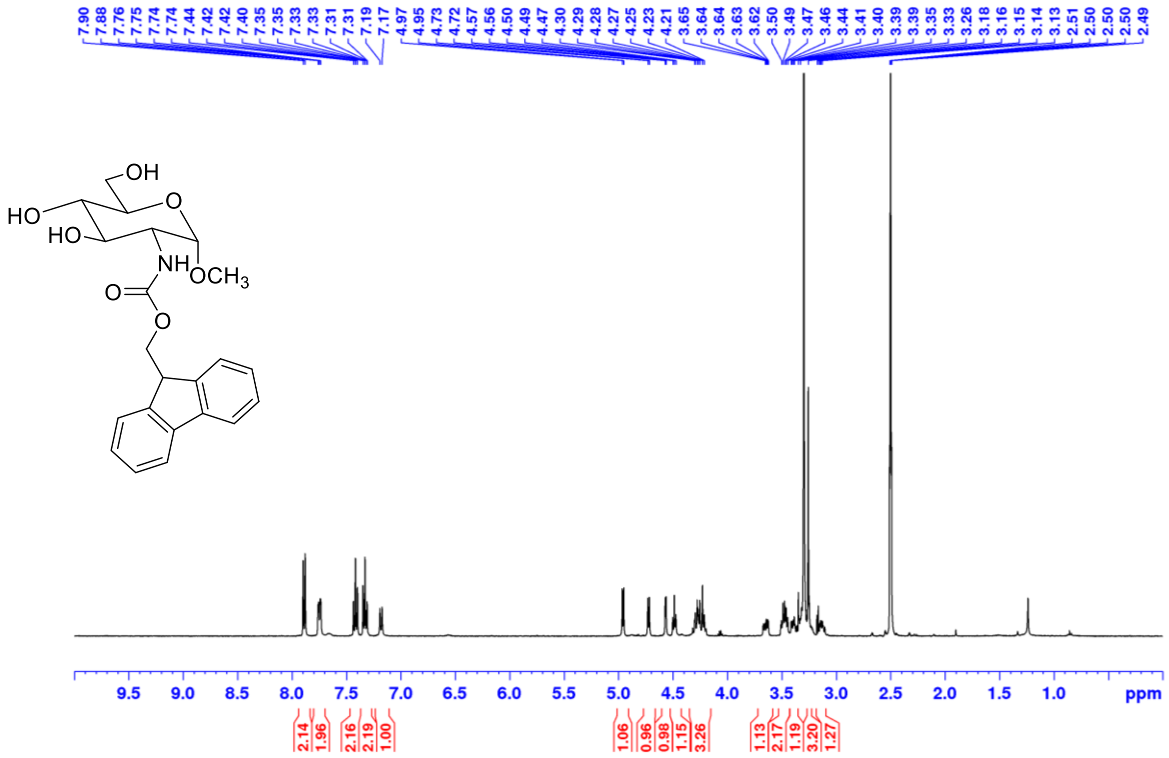

Figure S19. ^{1}H NMR spectrum of compound **21** in d6-DMSO.

Part II. Additional Experiment for Drug Delivery Studies

1. Chloroquine Diphosphate Trapping and Release

Calibration Curve

A serial dilution was performed using chloroquine diphosphate salt (Acros, CAS: 50-63-5) to obtain the standard concentration calibration curve of the drug. An aqueous stock solution was prepared by dissolving chloroquine diphosphate salt (2.0 mg) in DI water in a 10 mL volumetric flask to obtain a concentration of 0.388 mM (0.2 mg/mL). This was then serial diluted by transferring 5.0 mL of the solution to a 10 mL volumetric flask using a volumetric pipette, then diluting it with DI water to the graduation line. This procedure was repeated to obtain solutions with concentrations of 0.194 mM (0.1 mg/mL), 0.0969 mM (0.05 mg/mL), 0.0485 mM (0.025 mg/mL), 0.0242 mM (0.0125 mg/mL), and 0.0121 mM (0.00625mg/mL). The UV–Vis spectra of these solutions and the concentration calibration curve is shown in Figure S20.

McIlvaine pH buffer solutions were prepared to be used for the chloroquine drug release study. These solutions were prepared at pH 7.0 and pH 3.0 by mixing set volumes of 0.2 M sodium phosphate dibasic (JT Baker analyzed reagent, 5-3828) with 0.1 M citric acid (Thermo Fisher, CAS: 77-92-9). Reagent were purchased from Thermofisher USA.

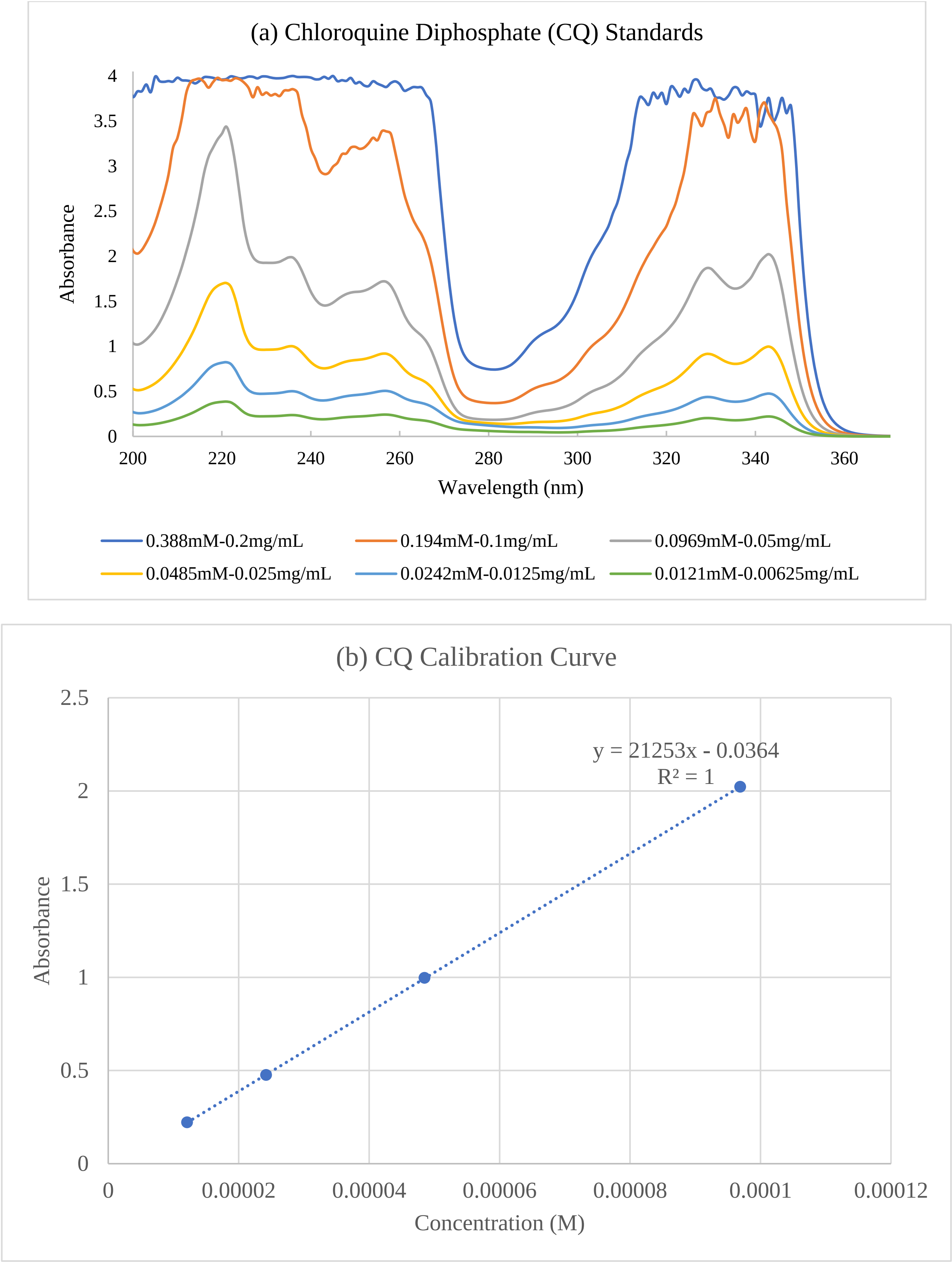

Figure S20. (a) UV–Vis spectra of chloroquine diphosphate dilution study, (b) Standard curve of chloroquine diphosphate (CQ).

Chloroquine Trapping and Release Studies at pH = 7

A stock solution was prepared by dissolving chloroquine diphosphate (1.0 mg) in DI H_2O (10.0 mL), then serial diluting ×2 to obtain a 0.0485 mM (0.025 mg/mL) solution. A 2 mL gel was prepared by heating compound **9** (4.0 mg) with chloroquine stock solution (2.0 mL, 0.05 mg) until the gelator fully dissolved. The sample was left to sit at room temperature for ~1 day. The gelator concentration was 2.0 mg/mL and chloroquine concentration was 0.025 mg/mL. The gel photos are shown below at time 0 h.

A pH 7 McIlvaine buffer (2.0 mL) was placed on top of the gel. At specific time intervals, all of the buffer was carefully removed and transferred to a quartz cuvette to record the UV absorbance. The supernatant was then returned to the gel vial at each time interval. The UV absorbance was recorded at 1 hr, 2 h, 4 h, 6 h, 8 h, 10 h, 24 h, 28 h, 32 h, 48 h, 72 h, and 145 h. The UV–Vis spectra are included in the manuscript Figure 7, and the estimated percent release bar graph is shown in Figure S21.

The molarity and percent release were calculated using the linear equation from the chloroquine diphosphate standard curve (y = 21253x - 0.0364). Approximately 51.6% of the chloroquine was released over 145 h.

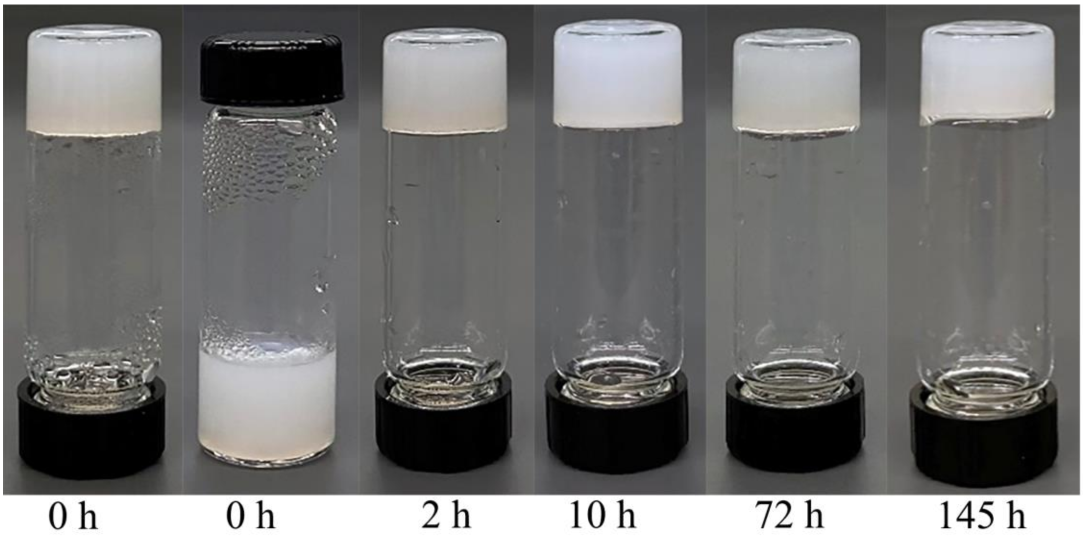

(**a**)

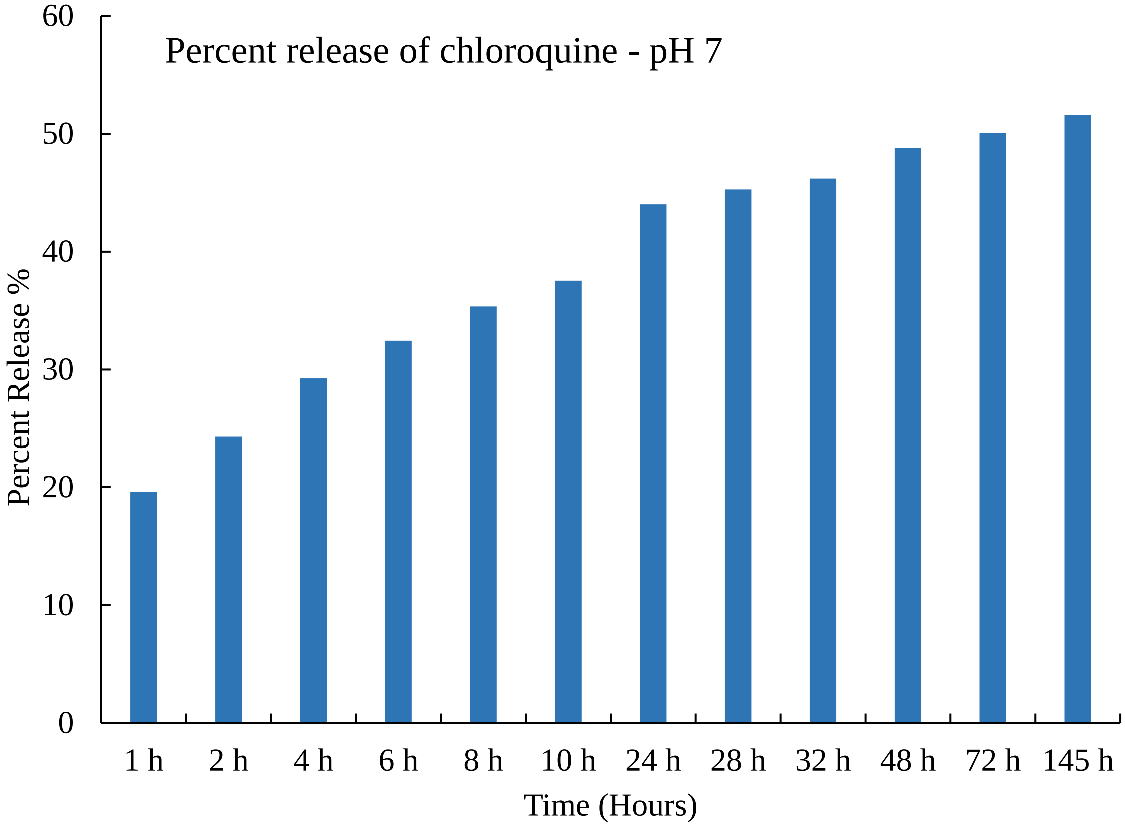

(**b**)

Figure S21. (**a**). Gel photographs of compound **9** with chloroquine at different time points after the pH=7 buffer was removed. (**b**) Percent release profiles of chloroquine diphosphate to a pH 7.0 aqueous phase over time from cogels formed by compound **9** (2.0 mg/mL gels). Percent release was calculated using absorption values at 343 nm for each time point versus the standard.

Chloroquine trapping and release studies at pH 3

A stock solution was prepared by dissolving chloroquine diphosphate (1.0 mg) in DI H_2O (10.0 mL), then serial diluting ×2 to obtain a 0.0485 mM (0.025 mg/mL) solution. A 2.0 mL gel was prepared by heating compound **9** (4.0 mg) with the chloroquine stock solution (2.0 mL, 0.05 mg) until the gelator was fully dissolved. The sample was left to sit at room temperature for ~1 day. The gelator concentration was 2.0 mg/mL and the chloroquine concentration was 0.025 mg/mL. The gel photos are shown below at time 0 h.

A pH 3 McIlvaine buffer (2.0 mL) was placed on top of the gel. At specific time intervals, all of the buffer was carefully removed and transferred to a quartz cuvette to record the UV absorbance. The supernatant was then returned to the gel vial at each time interval.

The UV absorbance was recorded at 1 h, 2 h, 4 hr, 6 h, 8 h, 10 h, 24 h, 28 h, 32 h, 48 hr, 72 h, and 145 h and included in Figure 8 in the manuscript.

The molarity and percent release were calculated using the linear equation from the chloroquine diphosphate standard curve (y=21253x-0.0364). Approximately 61.1% of the chloroquine was released over 145 h.

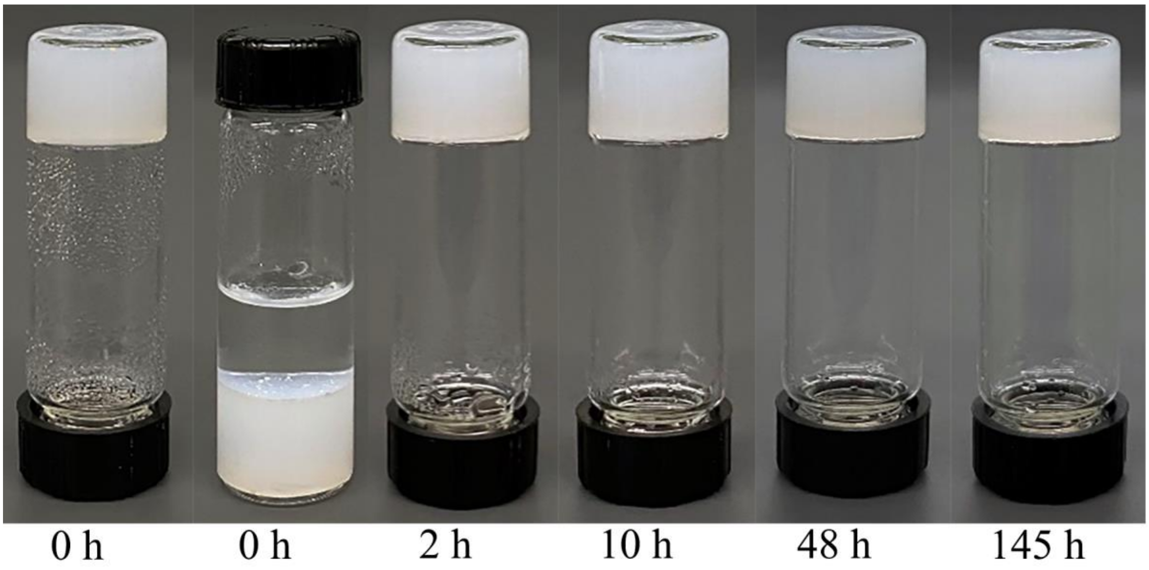

(**a**)

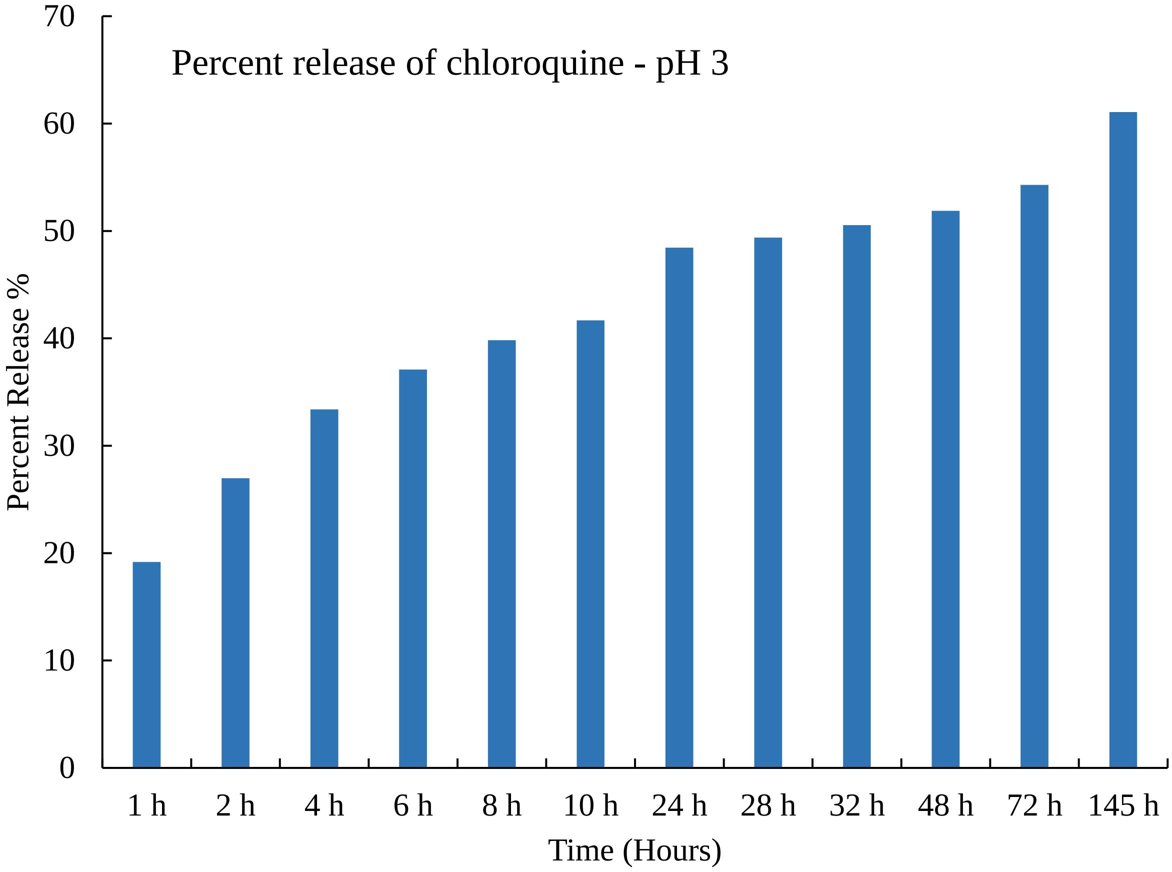

(**b**)

Figure S22. (**a**). Gel photographs of compound **9** with chloroquine at different time points after the pH=3 buffer was removed. (**b**). Percent release profiles of chloroquine diphosphate to a pH 3.0 aqueous phase over time from cogels formed by compound **9** (2.0 mg/mL gels). Percent release was calculated using absorption values at 343 nm for each time point versus the standard.

2. Naproxen Trapping and Release Studies

A total of three trials were conducted, and the results included in the main text are from Trial 3. The following sections include the details of the other two trials.

Trial 1

A stock solution was prepared by dissolving naproxen sodium (2.0 mg) in DI H_2O (2.0 mL). A 2 mL gel was prepared by heating compound **9** (4.0 mg) with naproxen sodium stock solution (0.5 mL, 0.5 mg) and DI H_2O (1.5 mL), which was left to sit at room temperature for ~6 days. DI H_2O (2.0 mL) was placed on top of the gel, with some of the water going underneath the gel. At specific time intervals, all of the water was carefully removed (including the water under the gel) and transferred to a quartz cuvette to record the UV absorbance. Due to the water going underneath the gel at multiple time points, these results are not an accurate representation of the drug diffusion. This trial result was not used for the study.

Trial 2

A stock solution was prepared by dissolving naproxen sodium (2.5 mg) in DI H_2O (10.0 mL). A 2 mL gel was prepared by heating compound **9** (4.0 mg) with naproxen sodium stock solution (2.0 mL, 0.5 mg, 0.25mg/mL), which was left to sit at room temperature for ~1 day. DI H_2O (2.0 mL) was placed on top of the gel, with some of the water going underneath the gel. At specific time intervals, all of the water was carefully removed (including the water under the gel) and transferred to a quartz cuvette to record the UV absorbance. The supernatant was then returned to the gel vial at each time interval. The UV absorbance was recorded at 1 hr, 2 hr, 4 hr, 6 hr, 8 hr, 24 hr, 28 hr, 32 hr, and 72 hr. The molarity and percent release were calculated using the linear equation from the naproxen sodium standard curve (Figure S7). Approximately 55% of the naproxen sodium was released over 72 hours. Due to the water going beneath the gel at multiple time points, these results are not an accurate representation of the drug diffusion. These are shown in Figures S23 and S24. The naproxen standard calibration spectra and curve are shown in Figure S25.

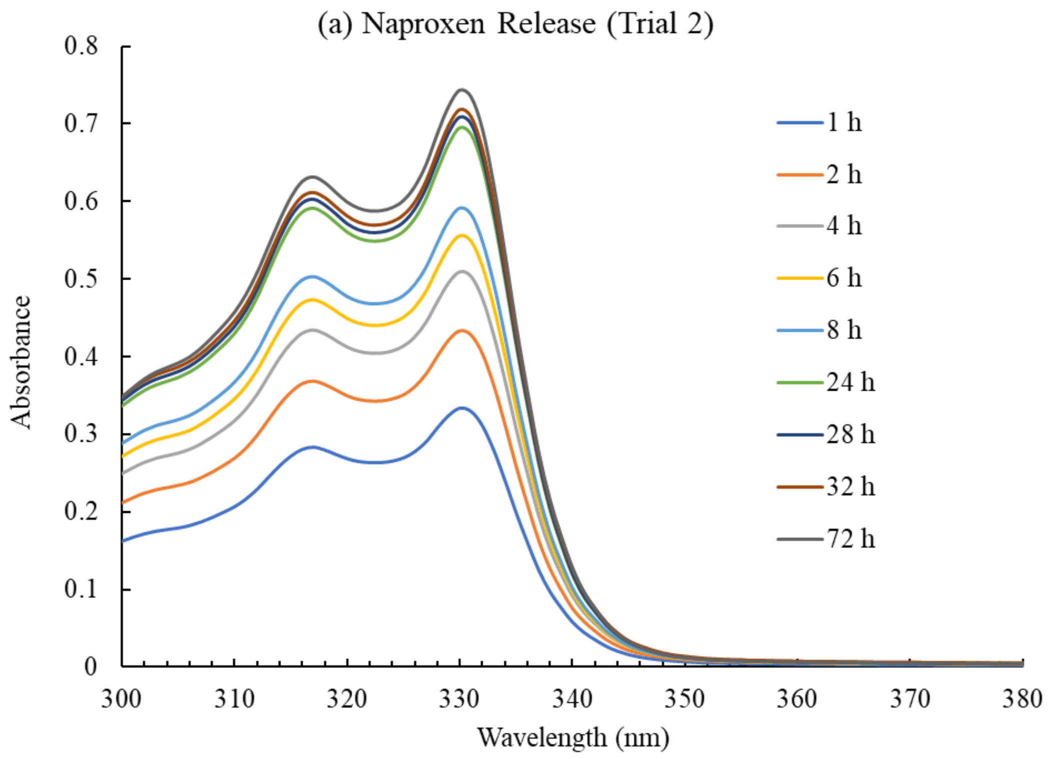

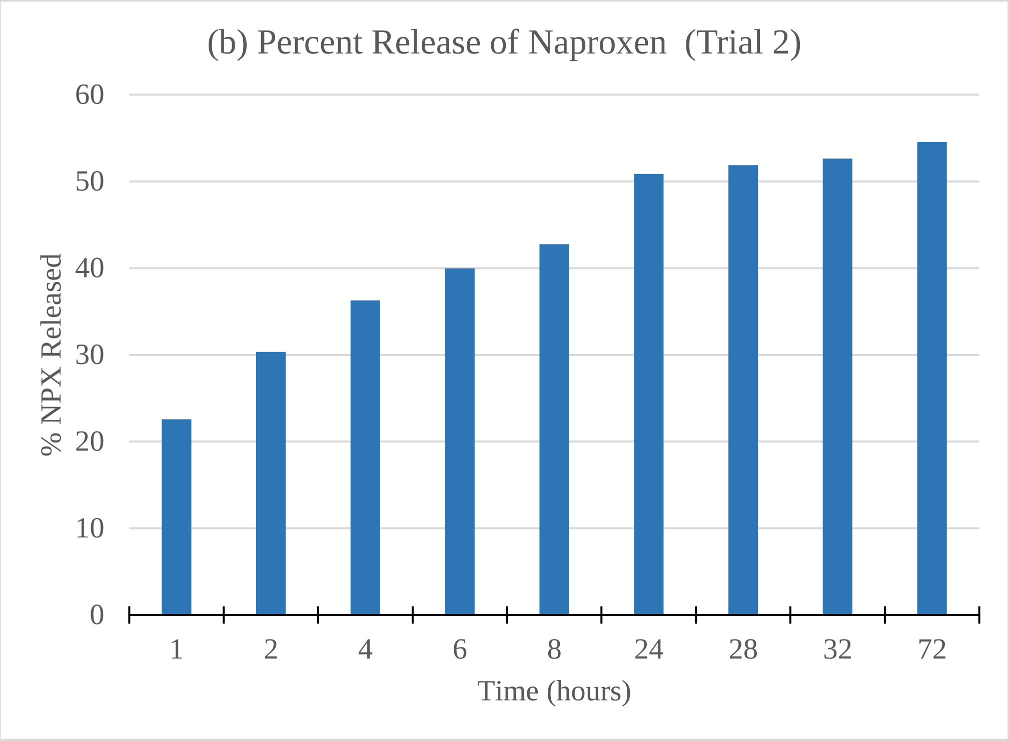

Figure S23. (a). UV–Vis spectra of naproxen sodium release study (Trial 2), (b) Percent release of naproxen sodium over time (Trial 2).

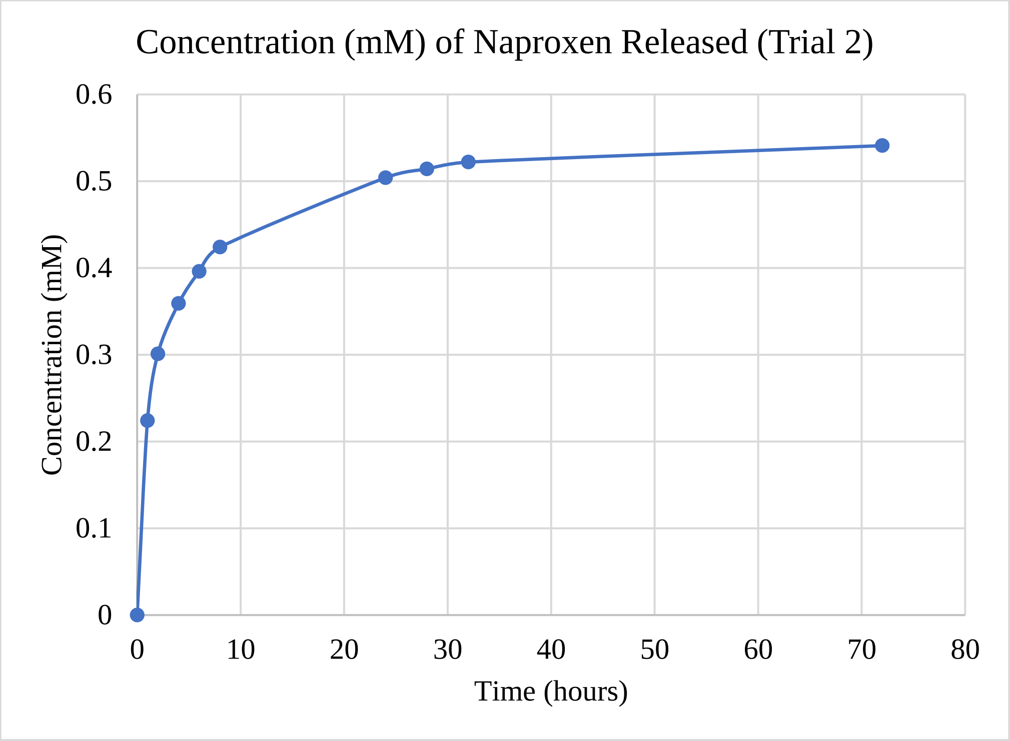

Figure S24. Concentration (mM) of naproxen sodium released over time (Trial 2).

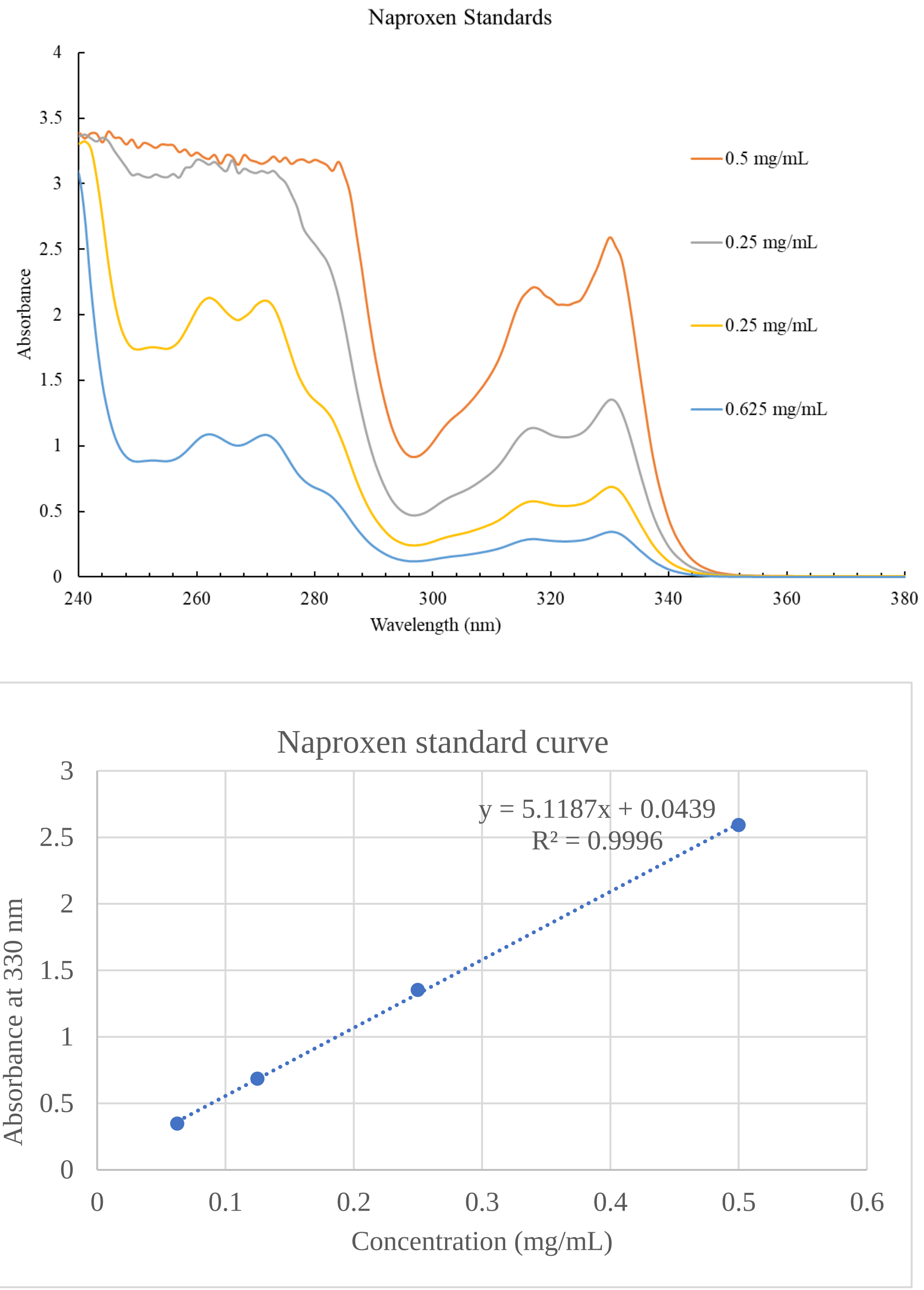

Figure S25. The naproxen standard calibration from the UV–Vis absorption.

3. Acid triggered gelation studies

Triggering studies were carried out in 1 dram vials. First, 2.0 mg of compound **10** was measured out and 0.2 mL of pH 1 solution (a) and pH 4 solution (c) was added. At room temperature, gelation did not occur after 24 hours; however, gelation was observed after gently heating the mixture and allow it to cool to rt.

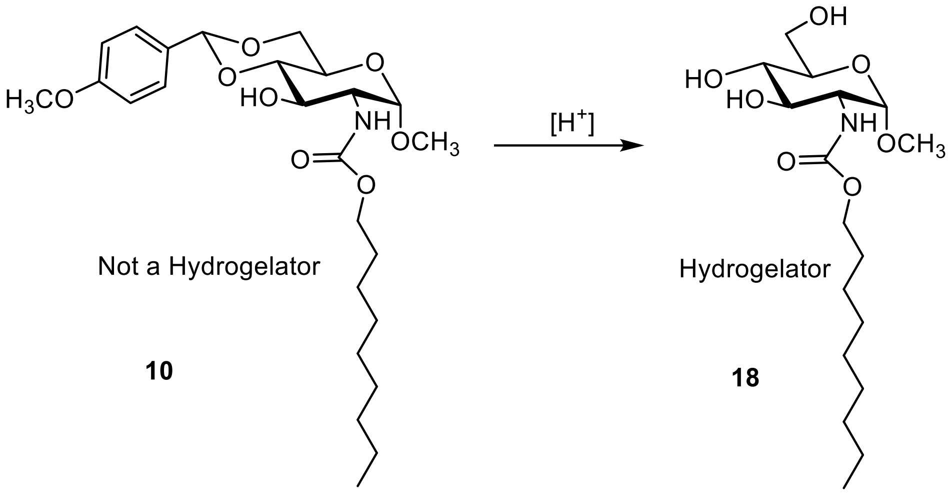

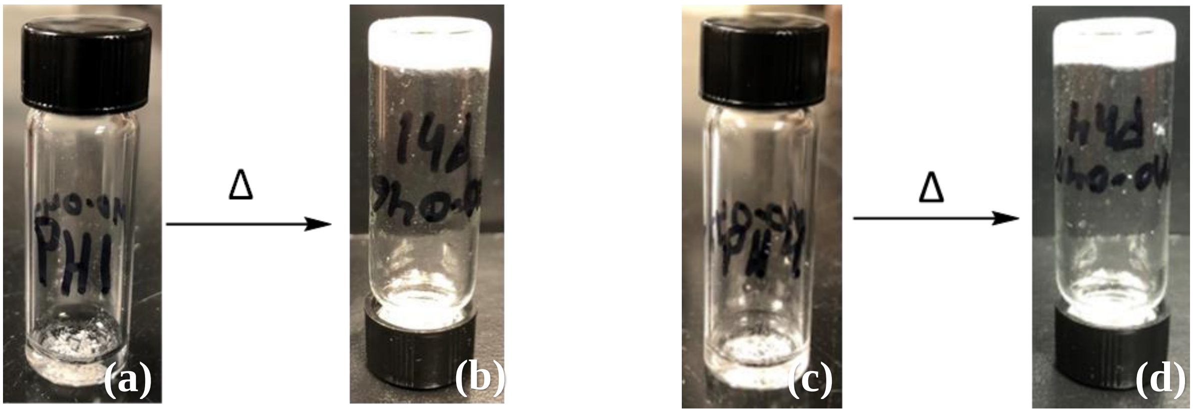

Figure S26. (**a**) Compound **10** and pH 1 aqueous solution mixture; (**b**) the sample in (a) after heating; (**c**) Compound **10** and pH 4 aqueous solution mixture; (**d**) the sample in (c) after heating.

In a repeated experiment, 4.0 mg of gelator **10** was added to a scintillation vial containing 0.4 mL of pH 1 solution. The vial was placed in a MaxZ 4000 incubator/shaker at 37 °C at 95 rpm; after 24 hours small globs of gel could be observed, and after 72 hours, all solid had disappeared. A sample was deposited onto a glass slide and observed under a microscope, the optical microscope image showed the molecules had formed self-assembly into fibers (Figure S27).

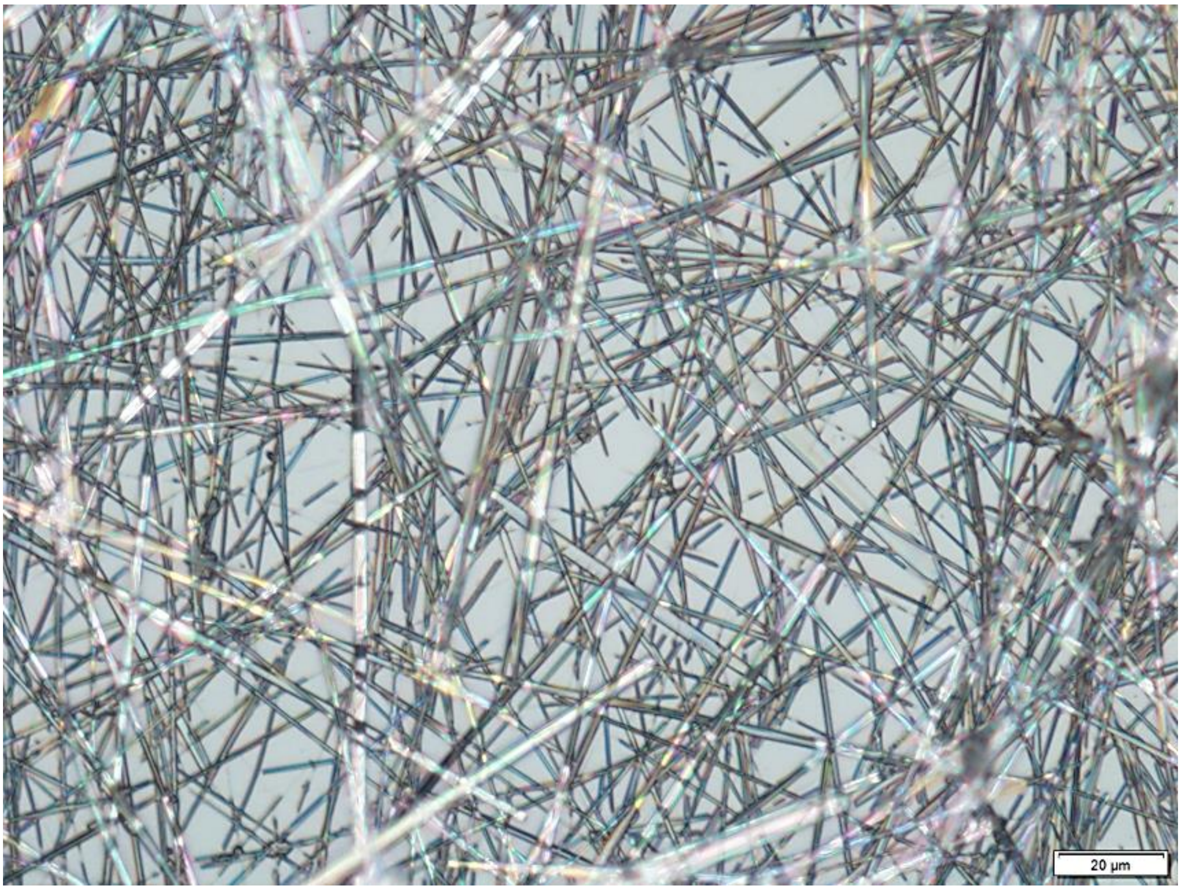

Figure S27. Optical micrograph of the sample from heating compound **10** in pH 1 solution after heating at 37 °C for 72 h.
